# A custom magnetoencephalography device reveals brain connectivity and high reading/decoding ability in children with autism

**DOI:** 10.1038/srep01139

**Published:** 2013-01-25

**Authors:** Mitsuru Kikuchi, Yuko Yoshimura, Kiyomi Shitamichi, Sanae Ueno, Tetsu Hirosawa, Toshio Munesue, Yasuki Ono, Tsunehisa Tsubokawa, Yasuhiro Haruta, Manabu Oi, Yo Niida, Gerard B. Remijn, Tsutomu Takahashi, Michio Suzuki, Haruhiro Higashida, Yoshio Minabe

**Affiliations:** 1 Research Centre for Child Mental Development, Kanazawa University, Kanazawa 920-8640, Japan; 2 Departments of Psychiatry and Neurobiology, Graduate School of Medical Science, Kanazawa University, Kanazawa 920-8640, Japan; 3 Higher Brain Functions & Autism Research, Department of Child Development, Osaka University United Graduate School of Child Development, Osaka University, Kanazawa University, Hamamatsu University School of Medicine, Chiba University, and Fukui University, Kanazawa 920-8640, Japan; 4 International Education Centre, Kyushu University, Fukuoka 815-8545, Japan; 5 Department of Anaesthesiology, Graduate School of Medical Science, Kanazawa University, Kanazawa 920-8640, Japan; 6 Department of MEG, Yokogawa Electric Corporation, Kanazawa 920-0177, Japan; 7 Department of Neuropsychiatry, School of Medicine, University of Toyama, Toyama 930-0194, Japan

## Abstract

A subset of individuals with autism spectrum disorder (ASD) performs more proficiently on certain visual tasks than may be predicted by their general cognitive performances. However, in younger children with ASD (aged 5 to 7), preserved ability in these tasks and the neurophysiological correlates of their ability are not well documented. In the present study, we used a custom child-sized magnetoencephalography system and demonstrated that preserved ability in the visual reasoning task was associated with rightward lateralisation of the neurophysiological connectivity between the parietal and temporal regions in children with ASD. In addition, we demonstrated that higher reading/decoding ability was also associated with the same lateralisation in children with ASD. These neurophysiological correlates of visual tasks are considerably different from those that are observed in typically developing children. These findings indicate that children with ASD have inherently different neural pathways that contribute to their relatively preserved ability in visual tasks.

Recent behavioural investigations have revealed that individuals with autism spectrum disorder (ASD) perform more proficiently on certain visual tasks (e.g., the visual reasoning task)^1^,^2^,^3^,^4^ than may be predicted by their general cognitive performances. No previous study has concentrated on the link between such innate abilities and objective brain function in young children with ASD, as this is a challenging age (<8) for performing brain imaging scans, such as functional magnetic resonance imaging, particularly under conscious conditions. To overcome these challenges, we developed a custom-made magnetoencephalography (MEG) device for young children (PQ 1151R model; Yokogawa/KIT Corp, Kanazawa, Japan) in which the MEG sensors are placed especially near to the head to allow for optimal recording in young children (Figure 1a)^5^,^6^. These benefits are not available when imaging young children using conventional, adult-sized MEG systems (Figure 1b). As little as 3 min is required to scan the brain's functional connectivity; therefore, this device enabled us to image conscious children with ASD. In the present study, we (i) analyse intrahemispheric coherence to determine the degree of phase-locking of neural activities across different brain regions (Figure 1c), (ii) investigate the brain neurophysiological profile that is associated with the preserved visual reasoning ability in children with ASD and typically developing (TD), and (iii) clarify the differences in the neural correlates between children with ASD and TD children.

Coherent brain rhythms represent a core mechanism for sculpting temporal coordination of neural activity across the brain-wide network^7^. Coherent oscillations participate in the well-timed coordination and communication between the neural populations that simultaneously engage in cognitive processes^7^. MEG produces a reference-free signal on a millisecond timescale and is an ideal tool for computing the coherence between two distant cortical rhythms. One critical limitation should be taken into account when considering the results of coherence analyses in neurophysiological studies: the coherence between a pair of sensors can increase if a single source generates a signal that reaches both sensors. In the present study, to reduce the probability of this occurrence, we used a sparse alignment of the MEG sensors. As is indicated in Figure S2, magnetic field strength diminishes approximately with the square of the distance from the source, and the sparse alignment of the MEG sensors (i.e., a long distance between the sensors) reduces the probability that a robust signal from a single source reaches multiple sensors. The MEG data were analysed off-line for 10 intrahemispheric coherences in both of the hemispheres. These measurements were made between the five regions of interest (Figure 1c)^5^.

The asymmetric processing of cognitive information has long been an intriguing property of the human brain^8^; however, the unusual lateralisation of brain function^9^,^10^ and anatomy^11^ is believed to be an early and invariant event, and a disruption in this process may reflect a core fundamental neurodevelopmental pathology in the subjects with ASD. Therefore, in addition to coherence values in each hemisphere, we examined the laterality indices (LIs) for each of the observed intrahemispheric coherences. These LIs were analysed as potential neurophysiological markers. The LIs were calculated using the following formula: LI = (L − R)/(L + R), where L and R represent the coherence values from the left and the right hemispheres, respectively.

## Result

A total of 26 TD children (22 males and 4 females) and 26 children with ASD (22 males and 4 females) participated in the study. The mean ages of the TD children and the children with ASD were 70.8 months (range: 60–82) and 71.3 months (range: 62–92), respectively. To assess general cognitive function in the participants, all of the children performed the Kaufman Assessment Battery for Children (K-ABC)^12^. The mean scores on the K-ABC mental processing scale were 99.9 ± 10.8 (mean ± SD) and 95.0 ± 19.2 in the TD children and the children with ASD, respectively. The parents agreed to allow their children to participate in the study and had full knowledge of the experimental nature of the research. Written informed consent was obtained prior to the beginning of the experiment. To assess visual reasoning performance, we used the modified scores from the K-ABC Matrix Analogies subtest. For comparison, we used the scores from the K-ABC Riddles subtest to assess verbal reasoning performance^12^.

### The profile of the K-ABC sub-scores in children with ASD and TD children

When we compared the performances on all of the K-ABC subtests between the TD children and the children with ASD (Figure S1), an unpaired *t*-test expectedly revealed that “Riddles” (i.e., the verbal reasoning task) was the weakest subtest for the children with ASD (df = 50, t = 3.01, *P* = 0.004) (Figure 2a). Alternatively, as expected, we observed no difference in the scores for “Matrix Analogies” (i.e., the visual reasoning task) between the TD children and the children with ASD (Figure 2a). Unexpectedly, the Reading/Decoding” (i.e., the reading ability task) performances tended to be higher in the ASD group than in the TD group (Figure 2a). Intriguingly, the variance of this score in the children with ASD (mean = 8.3, 1SD = 7.8) was larger than for the TD children (mean = 6.1, 1SD = 3.4). This variance was also larger than it was for the scores that the children with ASD obtained for the other K-ABC subtests, indicating that there is a greater degree of variability in the reading ability of the children with ASD than in TD children. This diversity is consistent with the general clinical reports that many conditions are comorbid with ASD (e.g., hyperlexia^13^, precocity, and mental retardation^14^).

### Preserved ability on visual tasks in children with ASD

As is indicated in Figure 2a, a two-way ANOVA (psychological performance × group) revealed a significant interaction (df = 2, F = 10.562, *P* < 0.0001). This result supported our hypothesis that young subjects with ASD achieve higher scores on visual tasks than would be expected based on their performances on the verbal task (Figure 2b, 2c). Certain children with ASD with low verbal reasoning test scores achieved high scores on pattern reasoning or reading/decoding tests. This result is comparable to those of past studies that examined individuals in higher age groups (i.e., aged 7 to 16)^2^.

### Pattern reasoning performance and intrahemispheric connectivity

To reveal the specific neurophysiological connectivity that is associated with higher performance in visual pattern reasoning in children with ASD and TD children, we used multiple linear regression analysis with the visual reasoning test scores and age (i.e., the two independent variables) to predict (i) intrahemispheric coherence in each hemisphere and (ii) the LI (i.e., the dependent variables). Due to the multiple comparisons in the 10 intrahemispheric pairs for the 9 frequency bands, our alpha level was adjusted to 0.05/90 = 0.00056 for the results of both the intrahemispheric coherence in each hemisphere and the corresponding LI.

First, to investigate the neurophysiological profile that is associated with higher visual reasoning ability regardless of the existence of a disorder, multiple linear regression analyses were applied for all of the participants (i.e., n = 52, children with ASD and TD children). The correlation coefficients between the two independent variables were 0.410 for all of the participants (n = 52). As is indicated in Tables S1–S3, the Matrix Analogies subtest score and age were not significant predictors of any intrahemispheric coherence in either hemisphere or of the corresponding LIs.

Second, to investigate the neurophysiological profile that is associated with the higher visual reasoning ability and which is specific for the children in the two groups, multiple linear regression analyses were separately applied for children with ASD (n = 26) and TD children (n = 26). The correlation coefficients between the two independent variables were 0.397 for the children with ASD and 0.455 for the TD children. The Matrix Analogies subtest score and age were not significant predictors of any intrahemispheric coherence in children with ASD (Figure 3a, b; Table S4, S5) or in TD children (Table S7, S8). As is shown in Figure 3c and Table S6, the Matrix Analogies subtest score (β = −0.641, *P* = 0.0004) and age (β = 0.682, *P* = 0.0002) were significant predictors of the LI in the gamma-2 band coherence (in the parieto-temporal (P-T)) for children with ASD. In TD children, these variables are not a significant predictor of any LI (Table S9).

### Reading ability and intrahemispheric connectivity

To reveal the specific neurophysiological connectivity that is associated with higher performance in the reading task, we used multiple linear regression with reading performance and age (i.e., the two independent variables) to predict intrahemispheric coherence in each hemisphere and the corresponding LIs (the dependent variables). As in the previous analysis, the alpha level was adjusted to 0.05/90 = 0.00056.

First, to investigate the neurophysiological profile that is associated with the higher reading ability regardless of the existence of a disorder, multiple linear regression analyses were applied for all of the participants (i.e., n = 52, children with ASD and TD children). The correlation coefficients between the two independent variables were 0.551 for all of the participants (n = 52). As is shown in Table S10–S12, the Reading/Decoding subtest score was not a significant predictor of any intrahemispheric coherence in either hemisphere or of the corresponding LIs. Age was a significant predictor for the LI of the P-T coherence in the gamma-2 band (β = 0.574, *P* = 0.0003) (Table S12).

Second, to investigate the neurophysiological profile that is associated with higher reading ability and specific to the children in the two groups, multiple linear regression analyses were applied separately for the children with ASD (n = 26) and the TD children (n = 26). The correlation coefficients between the two independent variables were 0.674 for the children with ASD and 0.373 for the TD children. In children with ASD, the Reading/Decoding subtest score was a significant predictor of the gamma-2 band coherence (in the P-T coherence) in the right hemisphere (β = 0.905, *P* = 0.0002) (Figure 4b, Table S14) and the corresponding LI (β = −0.820, *P* = 0.0002) (Figure 4c, Table S15). In TD children, this score was a significant predictor of the LI in the theta-2 band coherence (in the front-occipital (F-O)) (β = −0.685, *P* = 0.0003) (Table S18). This score was not a significant predictor of any other coherence or of the corresponding LI in either group (Figure 4a–c; Tables S13–S18). Age was a significant predictor only for the P-T coherence in the gamma-2 band in children with ASD (β = 0.980, *P* < 0.0001) (Table S15).

As a supplementary analysis of children with ASD, we performed an additional coherence analysis using a full set of MEG sensors (i.e., 151 channels). Sensors that corresponded to the right P-T, in which significant neuronal correlates were observed, were selectively used as seed sensors. We then calculated the coherence values between the seed sensor and the remaining 150 sensors in the gamma-2 band. To reveal the specific neurophysiological connectivity that is associated with higher performance in the reading task, we used multiple linear regression with reading performance and age (i.e., the two independent variables) to predict coherence with each seed sensor (i.e., the dependent variables). As is indicated in Figure 5a, when we used the right parietal region as a seed sensor, higher reading ability was a significant predictor of higher gamma-2 band coherence between the right parietal region (i.e., the seed sensor) and the right temporo-occipital region in children with ASD. As is shown in Figure 5b, when we used the right temporal region as a seed sensor, higher reading ability was a significant predictor of higher gamma-2 band coherence between the right temporal region (seed sensor) and the right frontal, the right parietal and the right temporo-occipital regions in children with ASD.

### Intrahemispheric connectivity in children with ASD and TD children

To compare the coherence values between the children with ASD and the TD children, we performed unpaired *t* tests. As in the previous analysis, the alpha level was adjusted to 0.05/90 = 0.00056. The unpaired t tests revealed significantly higher gamma-1 band coherence in the right hemisphere (temporo-occipital network) in the children with ASD compared with the TD children (t = 3.88, P < 0.00056) (Figure 6b, Table S19). There were no significant differences in the intrahemispheric coherences in the left hemisphere between the children with ASD and the TD children (Figures 6a, Table S19).

## Discussion

Recent developments in neuroimaging methods^15^ have demonstrated aberrant brain connectivity^15^,^16^,^17^,^18^,^19^,^20^ and/or aberrant lateralisation^9^,^10^,^20^,^21^ in individuals with ASD. However, our study is the first that focuses on the brain functional connectivity and its cognitive correlates in young children with ASD under conscious conditions.

Previous functional magnetic resonance imaging (fMRI) studies have reported that the neural substrates for visual reasoning exist in a widely distributed fronto-parietal network^22^,^23^,^24^,^25^,^26^. Intriguingly, one recent study of gifted and control adolescents demonstrated that the superiority in visual reasoning ability was driven not by additional cortical activation but by increased activation in the posterior parietal cortex, including the superior parietal lobule and the right intraparietal sulcus^24^. It can therefore be supposed that more efficient processing during visual reasoning tasks is associated with more localised (or independent) brain activity within posterior brain regions. Consistent with this assumption, we did not observed positive significant correlations between higher visual reasoning ability and anterior-posterior brain connectivity in either TD children or in children with ASD.

Using fMRI in healthy adult participants, a previous study focused separately on the neural correlates of visual analytic and figural reasoning tasks. This previous study demonstrated that analytic reasoning yielded (i) greater activation in the left than in the right hemisphere, and (ii) greater anterior than posterior activation. In contrast, figural reasoning yielded (i) greater activation in the right than in the left hemisphere and (ii) greater posterior than anterior activation^25^. The right posterior brain region is therefore crucial for figural reasoning abilities rather than analytic reasoning abilities. We find that rightward lateralisation of brain connectivity in posterior brain regions contributes to visual pattern reasoning in young children with ASD but not in TD children. This result suggests that individuals with ASD, who exhibit well-documented preservation in visual reasoning^1^,^2^,^3^,^4^,^26^, could use figure perceptual strengths during any visual reasoning task.

A recent fMRI study demonstrated the neural bases of the solving of visual reasoning task in adults and adolescents with ASD^26^. These authors demonstrated that subjects with ASD displayed relatively increased task-related activity in the posterior region (e.g., the occipital cortex) and decreased activity in the anterior region (e.g., the prefrontal cortex) during a visual reasoning task. Interestingly, these authors observed a trend towards increased activity in the right inferior parietal cortex in individuals with ASD during visual reasoning tasks. It has also been reported that the cognitive strategies that are adopted by individuals with ASD are different from those that are used by TD subjects during other visual tasks. For example, subjects with ASD exhibit right lateralised brain activity during a visual search task^27^, greater posterior brain activity during an embedded figures task^28^,^29^ and right lateralised brain connectivity during an n-back visual working memory task^21^. In conjunction with our results, it can be postulated that a functional preference for the right-posterior brain may play a prominent role during various visual tasks in individuals with ASD.

Language and visual search tasks are generally thought to activate the left and the right hemisphere, respectively. In addition, recent studies have reported that the development of language^5^,^30^ and visual search abilities^30^ is accompanied by the leftward and rightward lateralisation of brain activities, respectively. With regard to visual letter perception, a recent study demonstrated that invariant representations of letter identities are generated in the visual word form area (VWFA), which can be reproducibly identified in the left occipito-temporal sulcus^31^. The VWFA then projects to structures that are involved in phonological or lexico-semantic processes in the left hemispheric language area^31^,^32^,^33^. In contrast, a controlled, longitudinal training study of young non-reading kindergarten children demonstrated the initiation of both the right and left occipito-temporal cortex sensitivity to printed letters^34^. This result suggests that the right-hemispheric analogue of the VWF (R-VWFA) also contributes to the ability of young children to read letters. Intriguingly, in cases of lesion of the VWF in adults, the R-VWFA was reported to participate in residual reading abilities (e.g., letter-by-letter reading)^31^. Letter-by-letter reading implies that the normal ability to identify letter strings in a quasi-parallel fashion is lost. Patients who read letter-by-letter can decode the letters but fail to recognise letter strings as whole words using lexico-semantic processes. Given that right posterior brain regions, including the R-VWFA, do not have a practical role in higher level language processes but play a certain role in letter decoding, the observed right-posteriorly weighted brain function in individuals with ASD may be unsuitable for practical language processes. Consistent with this assumption, the group of children with ASD with normal fluid intelligence in the present study tended to exhibit a preserved ability on simple reading/decoding tasks but a significantly lower ability in verbal reasoning tasks. Furthermore, language impairment is one of the key features of ASD, and the failure to develop sophisticated language is one of the earliest signs of ASD^35^,^36^.

Theta-band oscillations have been believed to represent ongoing cognitive processes during memory tasks^7^. In addition, long-distance connectivity in the right hemisphere via theta band oscillation is believed to play a significant role in ongoing cognitive processes, especially during visual memory^37^,^38^,^39^. In the present study, TD children with right lateralised anterior-posterior long-distant connectivity via theta band oscillations may have had a certain degree of cognitive strength in figural memory. Therefore, such individuals may exhibit higher letter reading performances. In contrast, in children with ASD, the strength of the anterior-posterior connectivity via theta band oscillations was not correlated with letter-reading ability. This result was consistent with the aforementioned assumption that individuals with ASD tend to make use of right-posterior regions (i.e., more localized region) during various visual processes.

Gamma-frequency oscillations between neural networks are essential for cortical information processing^7^,^40^, and oscillations in posterior regions are believed to represent ongoing cognitive processes during visual perception and attention^41^,^42^,^43^,^44^,^45^,^46^,^47^,^48^. Accordingly, the topographical distribution of the gamma band network in posterior regions during the watching of a video programme may represent the individual features of the brain network that are involved in the visual processes of daily life. In the present study, direct comparison between the ASD and TD groups demonstrated that the brain connectivity via gamma oscillations is higher in children with ASD in posterior region (i.e., temporo-occipital connection in the right hemisphere) (Figure 6b). Furthermore, gamma band oscillation-induced right-posteriorly weighted brain connectivity was associated with visual reasoning (Figure 3c) and letter-reading ability (Figure 4b, 4c) in children with ASD but not in TD children. This result was consistent with the aforementioned assumption that individuals with ASD tend to make use of right-posterior regions during various visual processes.

**Figure 1:**
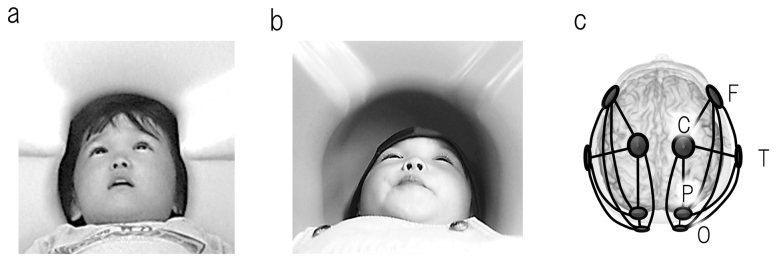
(a) A custom, child-sized MEG. (b) A conventional, adult-sized MEG. (c) The schema of the five selected sensors and the 10 intrahemispheric connections of interest in each hemisphere. F = selected sensor in frontal region; C = central; P = parietal; O = occipital; and T = temporal.

**Figure 2:**
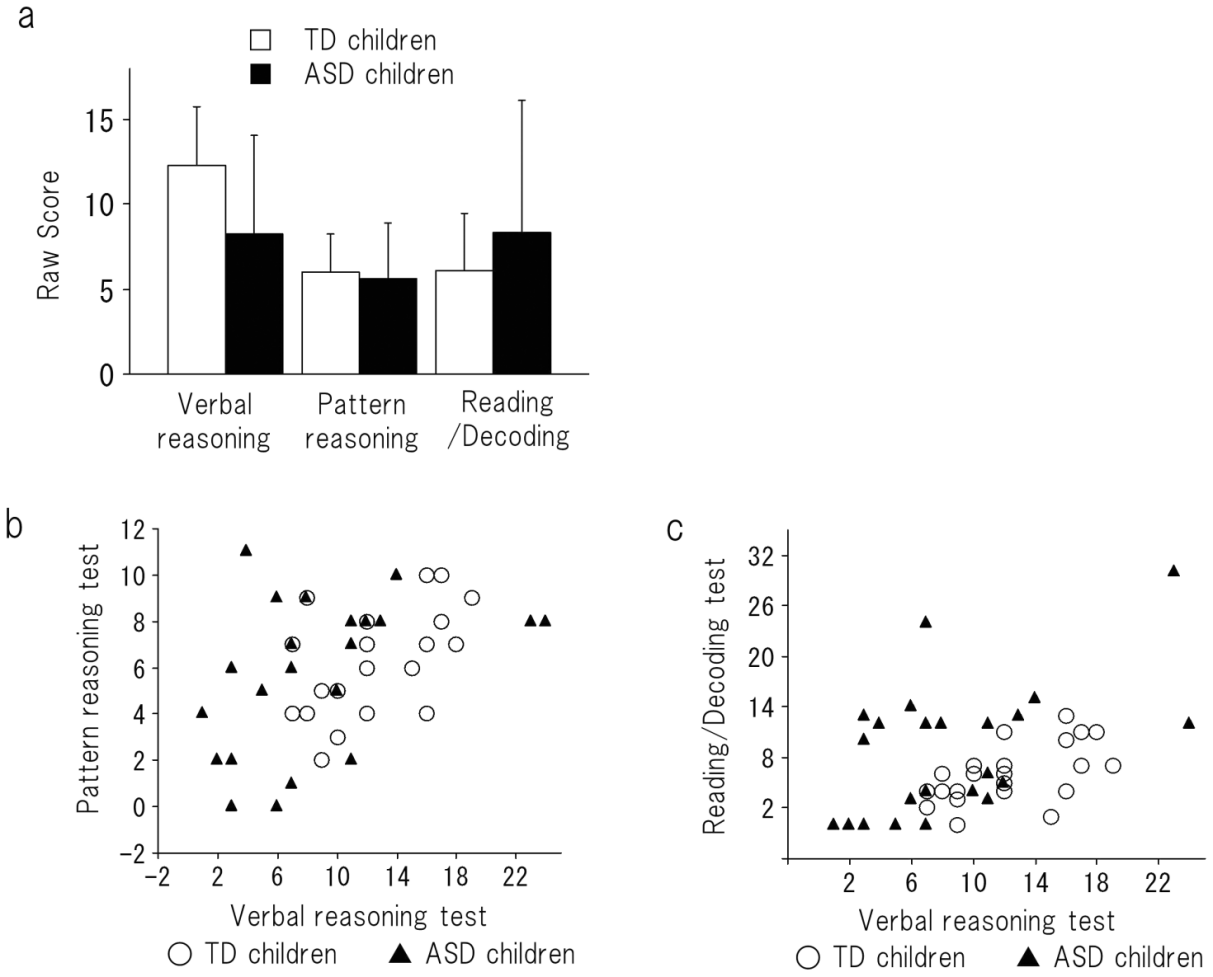
(a) The performance of young children with ASD and TD children on the verbal reasoning test, the pattern reasoning test and the reading/decoding test. The error bars represent 1 standard deviation. (b) A scatter plot is depicted of the two scores (i.e., for the verbal reasoning test and the pattern reasoning test) for the children with ASD and the TD children. (c) A scatter plot is depicted of two scores (i.e., the verbal reasoning test and the reading/decoding test) for the children with ASD and the TD children. Certain children with ASD with low verbal-reasoning test scores achieved relatively high scores on pattern reasoning or reading/decoding tests.

**Figure 3:**
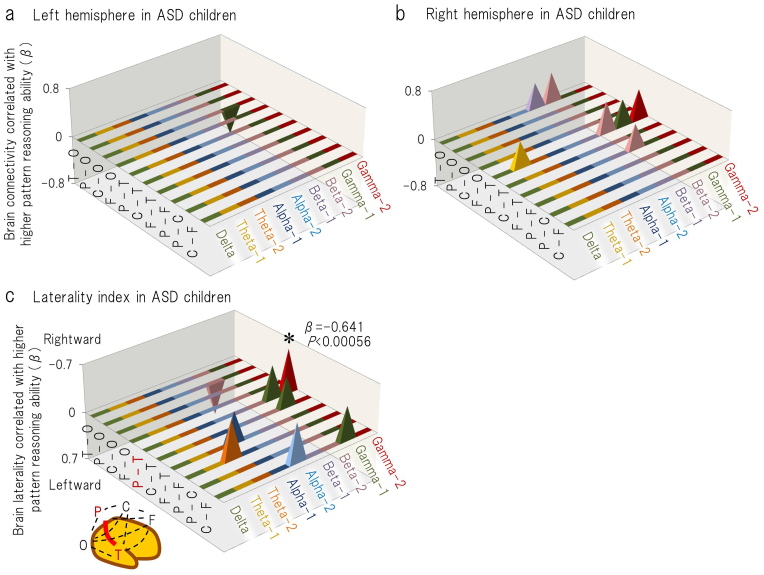
The standardised regression coefficients (β values) are shown for the pattern reasoning performance (one of the two independent variables) in the multiple regression model that was used to predict (a) left, (b) right intrahemispheric coherence and the corresponding laterality index (c) (i.e., the dependent variables), using age as the other independent variable, in the children with ASD. In sub-figures (a) and (b), the upward direction (positive β value) signifies a positive correlation between the pattern reasoning ability and the coherence value. In sub-figure (c), the upward direction (negative β value) signifies a positive correlation between the pattern reasoning ability and the rightward brain laterality. The β values are presented for *P* < 0.05. F = selected sensor in the frontal region; C = central; P = parietal; O = occipital; and T = temporal. *P < 0.00056.

**Figure 4:**
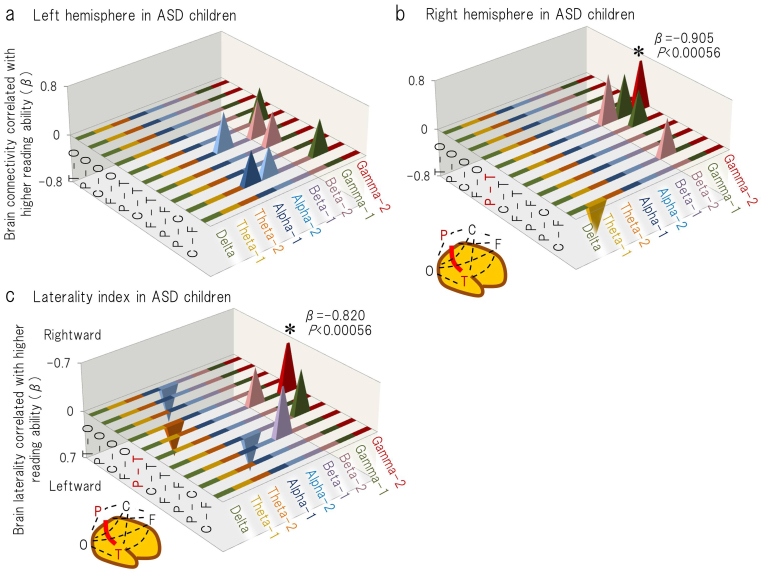
The standardised regression coefficients (β values) are shown for reading ability (one of two independent variables) in the multiple regression model that was used to predict (a) left, (b) right intrahemispheric coherence and the corresponding laterality index (c) (i.e., the dependent variables), with age as the other independent variable, in children with ASD. In sub-figures (a) and (b), the upward direction (positive β value) signifies a positive correlation between the reading ability and the coherence value. In sub-figure (c), the upward direction (negative β value) signifies a positive correlation between the reading ability and the rightward brain laterality. The β values are presented for *P* < 0.05. F = the selected sensor in frontal region; C = central; P = parietal; O = occipital; and T = temporal. *P < 0.00056.

**Figure 5:**
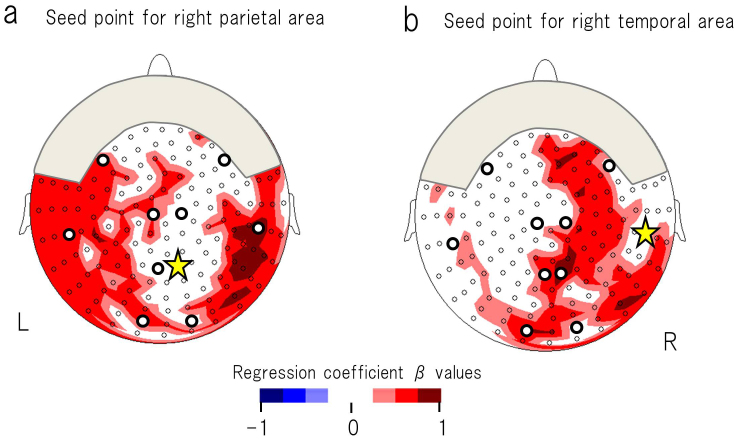
The standardised regression coefficients (β values) are topographically indicated. These values were calculated for reading ability (i.e., one of the two independent variables) to predict gamma-2 band coherence between the seed sensor and the remaining 150 sensors (i.e., dependent variables) in children with ASD. (a) A seed sensor was selected in the right parietal region. Higher reading ability was a significant predictor of higher coherence between the right parietal region (seed sensor) and the right temporo-occipital region. (b) A seed sensor was selected in the right temporal region. Higher reading ability was a significant predictor of higher coherence between the right temporal region (seed sensor) and the right frontal, the right parietal and the right temporo-occipital regions. The β values are presented for *P* < 0.05. The yellow star indicates the seed sensor. The open circles indicate the remaining 150 sensors. The open circles with bold lines indicate the sparse alignment of the MEG sensors for the first analysis in the present study. L = left; R = right.

**Figure 6:**
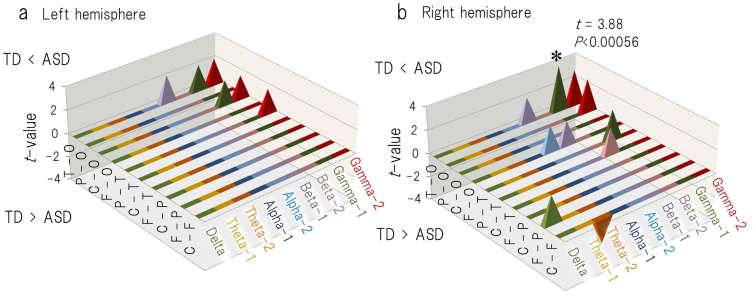
The t-values of intrahemispheric coherence for the (a) left and (b) right hemispheres between children with ASD (n = 26) and TD children (n = 26). Positive values represent greater levels in the ASD group than in the TD group. The t-values are presented for P < 0.05. F = selected sensor in the frontal region; C = central; P = parietal; O = occipital; and T = temporal. *P < 0.00056.

Although MEG is not the only child-friendly imaging technology, it is beneficial in studies that investigate the laterality of cortical oscillations. This benefit lies in the fact that magnetic fields that are generated unilaterally tend to reflect to sensors in the ipsilateral hemisphere. Furthermore, our new device (a custom-made MEG system for children) simultaneously recorded the right and the left hemispheres in young children (Figure 1a). This result would have been difficult to achieve had a conventional, adult-sized MEG system (Figure 1b) been used given the small head size of children relative to the sensor array. This study is the first to indicate that brain functional connectivity in the right hemisphere and/or rightward lateralisation in posterior brain regions contribute to natural reading and/or visual pattern reasoning abilities in young children with ASD. Our results suggest that the observed functional connectivity in the right hemisphere and the rightward lateralisation of intrahemisheric connectivity in posterior brain regions was associated with the preserved visual task performance in children with ASD. In addition, this contribution of rightward lateralisation to cognitive performance diminished with age, suggesting a delayed maturation of certain unspecified neural pathways in children with ASD. We emphasise that it is time for a new era of insight into the brains of younger children with ASD under conscious conditions.

Converging evidence suggests that the properties of gamma oscillations are altered in ASD during information processing^49^,^50^,^51^,^52^. In children with ASD (aged 3 to 8 years), one previous study demonstrated that an excess of EEG gamma band oscillations during sustained visual attention is directly related to the degree of developmental delay^49^. This altered gamma-band activity that was observed in young children with ASD may result from disturbances in GABAergic or glutamatergic mediator systems, which are critically important to generate this type of oscillation^53^, and suggested that changes in the excitation/inhibition balance may be a pervasive feature of cortical networks in ASD from a young age^54^. There are two possibilities to explain our results (an altered contribution of brain connectivity to cognitive performance in ASD). The first explanation is that we observed an ongoing aberrant excitation/inhibition balance, which brought on the diversified cognitive ability in young children with ASD. The second explanation is that an aberrant excitation/inhibition balance during the foetal period results in aberrant prenatal and perinatal development that leads to lasting alterations in neural networks.

The present study had several limitations. First, we could not evaluate the degree to which the subjects attended to the auditory or visual information in the video programmes. The children with ASD might have attended to the visual information rather than to the narrative sound information. Differences in these modality-dependent preferences may be reflected in the functional brain connectivity. Second, we eliminated any contaminated MEG data, such as when clear ocular movement occurred. However, differences in the fine ocular movements between the children with ASD and the TD children could have confounded the results when frequent saccades occurred, as may have happened during the watching of the video programme. Third, we recorded the head positions of the subjects using video monitors during the MEG recordings, and we eliminated any MEG data by visual inspection for coherence analysis if the head position of the subject had obviously moved from its starting position. Further study using a quantification algorithm for the head movement will provide more reliable evidence. Fourth, more than 40-sec periods were recommended when computing coherence^55^; however, after eliminating the contaminated data in the present study, the recording period that was accepted for analysis tended to be short (a minimum of 35 sec). In addition, the reliability of coherence in the gamma band is reported to be lower than that in the alpha band^55^, and these coherences could be significantly affected by the mental state of the subjects^56^. Further studies that employ longer periods and attention-controlled conditions will provide more reliable evidence, although these conditions will be difficult to achieve in conscious young children. Fifth, given that young children were examined in the present study, we were unable to obtain brain structural information on which to superimpose the coordinate systems of the source-estimated MEG signals. This limitation was encountered because it is troublesome, especially when studying young children, to perform additional MR imaging. We therefore performed sensor-level analysis instead of voxel-based analysis. To deduce the anatomical locations of the signal sources and to draw definitive anatomical conclusions, it will be necessary to perform further studies that use source localisation methods in combination with individual brain structural information.

## Methods

### Subjects

The clinical group included 26 children with autism spectrum disorder (22 males and 4 females). The subjects were aged 62–92 months and were recruited from the Kanazawa University Hospital and prefectural hospitals in Toyama. The children were diagnosed by a clinical psychiatrist and a speech therapist with more than 5 years of experience in ASD using the Autism Diagnostic Observational Schedule, Generic (ADOS)^57^, the Diagnostic Interview for Social and Communication Disorders (DISCO)^58^, and the DSM-IV criteria^59^ at the time of MEG and K-ABC data acquisition. All of the children with ASD who were included in the present study fulfilled the diagnosis of childhood autism (n = 18), atypical autism (n = 3) or Asperger's syndrome (n = 5) using the DISCO. The children below the ADOS cut-offs were included in the present study if they met the criteria for children with ASD using both the DSM-IV and the DISCO (i.e., 4 of 26 children). The controls were 26 typically developing children (22 males and 4 females) who were between 60 and 82 months of age with no reported behavioural or language problems. The control children were approximately matched to the subjects with autism with respect to age. All of the typically developing children were native Japanese and had no previous or existing developmental, learning, or behavioural problems according to the information that was obtained by a questionnaire, which was completed by their parents. All of the participants had normal hearing ability according to the available medical records. The dominant hands were determined by the children's preference when handling a spoon; the majority of the children were right-handed (TD children: right = 25, left = 1, both = 0; children with ASD: right = 20, left = 2, both = 4). All parents agreed to allow their children to participate in the study with full knowledge of the experimental nature of the research. Written informed consent was obtained before the start of the experiment, and the Ethics Committee of Kanazawa and Toyama University Hospital approved the methods and procedures that were used.

### MEG recordings

All of the children participated in the cognitive tasks and the MEG measurements on two separate days. On both of the testing days, female staff members made contact with the children and played with them along with their parent(s)/caretaker(s). During the MEG recording, one staff member (author YY) escorted each participant into the shielded room (Daido Steel, Nagoya, Japan), which was decorated with colourful pictures of Japanese cartoon characters and mimicked an attractive vehicle adopted from an animated series that is popular with preschool children. During the measurements, the staff member remained in the shielded room to comfort and encourage each participant to maintain a steady body position when necessary. The parent(s)/caretaker(s) could observe their children through a TV monitor during the measurements. According to their evaluations, none of the participants endured high emotional tension or other discomfort during the measurements. The MEG data were recorded using a multichannel SQUID (Super-conducting Quantum Interference Device), whole-head coaxial gradiometer MEG system for children. The MEG data were acquired using a sampling rate of 1000 Hz and were filtered using a 200-Hz low-pass filter. The children were supine on a bed during the MEG recording and viewed a video programme that was projected onto a screen. During the MEG recording time, we determined the head position within the helmet by measuring the magnetic fields that were generated by passing currents through coils that were attached at three locations on the surface of the head (as fiduciary points) in relation to the landmarks (the bilateral mastoid processes and the nasion). Prior to the recording session, we prepared several video programmes with stories that were particularly entertaining for young children. The video programme was selected according to the preference of each participant. The narration sound was binaurally delivered to the participants through a tube (leading to speakers that were placed outside of the shielded room) that was placed in front of the subject. We ensured that each subject was satisfied with the video programme that was selected prior to the recording.

### MEG data analysis

In the present study, 10 intrahemispheric coherences were measured for each hemisphere between the following five regions of interest: 1) frontal (F), 2) central (C), 3) temporal (T), 4) parietal (P) and 5) the occipital region (O) (Figure 1c). A selection of sensors that correspond to these brain regions was performed prior to the offline coherence analysis (as described in our previous report)^5^. An off-line analysis of the MEG data was performed using the Brain Vision Analyser (Brain Products, Gilching, Germany). The process of eliminating any contaminated data was blind to the personal data. A minimum 35-sec recording period was accepted for each subject. The MEG spectra were calculated using a Fast Fourier Transform. The coherence values were grouped into the following 9 bands: 1) delta (0.7–3.9 Hz); 2) theta-1 (4.2–5.9 Hz); 3) theta-2 (6.4–7.8 Hz); 4) alpha-1 (8.3–9.8 Hz); 5) alpha-2 (10.0–12.0 Hz); 6) beta-1 (12.2–19.8 Hz); 7) beta-2 (20.0–29.8 Hz); 8) gamma-1 (30.0–57.9 Hz) and 9) gamma-2 (62.0–79.8 Hz).

### Psychological tasks

#### Kaufman Assessment Battery for Children (K-ABC)

The children performed the Japanese adaptation of the Kaufman Assessment Battery for Children (K-ABC).

#### Visual reasoning task

We employed the modified scores of the Matrix Analogies subtest in the K-ABC^12^ to represent visual reasoning performance. The Matrix Analogies test requires the selection of a picture or design that best completes a visual scene or the pattern. This test is similar to Raven's Progressive Matrices test^60^, which is a nonverbal measurement test, as the examiner conveys the instructions through gestures, and the child responds with movements. We excluded the scores of the first three questions in the Matrix Analogies subtest, as they did not involve geometric patterns.

#### Verbal reasoning task

We employed the Riddles subtest scores in the K-ABC to compare verbal reasoning performances^12^. In the Riddles subtest, the children were required to retrieve a target word that was based on suggested words that were conceptually associated with the target word. This subtest generally evaluates the development of conceptual formation and conceptual inference as well as word retrieval abilities.

#### Reading ability task

The K-ABC Reading/Decoding subtest was used to evaluate the single-word reading ability of the children.

## Author Contributions

Authors Mitsuru Kikuchi, Haruhiro Higashida and Yoshio Minabe designed the study and wrote the protocol. Authors Tetsu Hirosawa, Yuko Yoshimura, Tsunehisa Tsubokawa, Yasuhiro Haruta and Gerard B. Remijn managed the literature searches and analyses. Authors Sanae Ueno and Kiyomi Shitamichi undertook the statistical analysis, and author Mitsuru Kikuchi wrote the first draft of the manuscript. Authors Yasuki Ono, Yuko Yoshimura, Manabu Oi, Toshio Munesue, Yo Niida, Tsutomu Takahashi and Michio Suzuki recruited subjects and assessed their symptom. All authors contributed to and have approved the final manuscript.

## Supplementary Material

Supplementary Information Supplement tableS1-S19 and figuresS1
