# Supplementary material for: A custom magnetoencephalography device reveals brain connectivity and high reading/decoding ability in children with autism
Source: Sci Rep. 2013 Jan 25;3:1139. doi: 10.1038/srep01139 (PMC3555087; doi:10.1038/srep01139)
Supplement: Supplementary Information — Supplement tableS1-S19 and figuresS1 [file srep01139-s1.doc]

Scientific Reports　(Research Reports)

**Supplemental information**

**Title: A custom magnetoencephalography device reveals brain connectivity and high reading/decoding ability in children with autism**

**Figure S1**


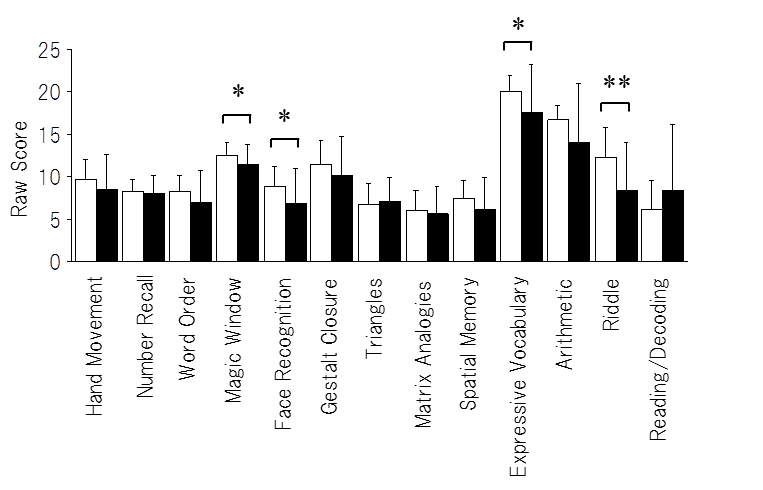


Figure S1. The performance of each K-ABC subtest in children with ASD and TD young children is shown. The error bars represent 1 standard deviation. An unpaired *t*-test revealed a significantly lower performance in children with ASD compared with TD children. **P*<0.05. ***P*<0.005. Note that the performance on “Reading/Decoding” (i.e., the reading ability task) was often higher in the ASD group. The variance of this score in the children with ASD (mean=8.3, 1SD=7.8) was greater than it was in the TD children (mean=6.1, 1SD=3.4) and those of other K-ABC subtests in children with ASD, which indicates a greater diversity of reading ability in children with ASD.

**Figure S2**


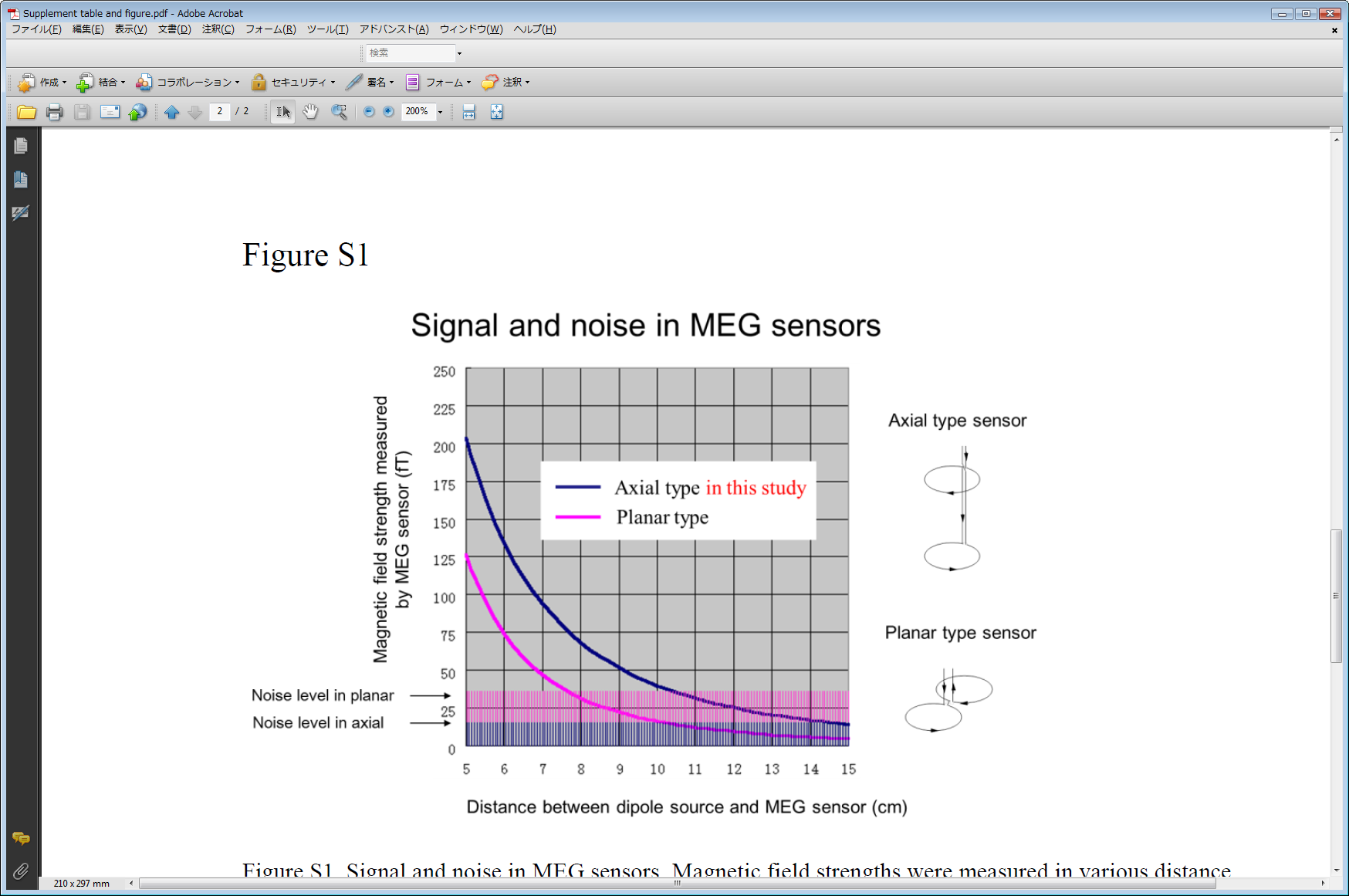


Figure S2, Signal and noise in MEG sensors. Magnetic field strength diminishes with the distance from the source. Magnetic field strengths were measured in various distance between dipole source and two types of MEG sensor. Experimental dipole source generated 10 nAm and was measured for 100 times for each distance. Signal and noise were evaluated based on the averaged data. Both axial and planar type sensors demonstrated extremely poor signal to noise ratio when distances were over 10 cm.

**Tables S1-S19**

Table S1. Correlations between coherences in left hemisphere and pattern reasoning ability calculated for all children (*n*=52, ASD and TD children). The standardised regression coefficient β values for the multiple regression model are shown with intrahemispheric coherences in left hemisphere. The Matrix Analogies subtest of the K-ABC and age were applied as independent variables.

| Matrix Analogies | Delta | Theta-1 | Theta-2 | Alpha-1 | Alpha-2 | Beta-1 | Beta-2 | Gamma-1 | Gamma-2 |
| --- | --- | --- | --- | --- | --- | --- | --- | --- | --- |
| T - O | n.s. | n.s. | n.s. | n.s. | n.s. | n.s. | n.s. | n.s. | n.s. |
| P - O | n.s. | n.s. | n.s. | n.s. | n.s. | n.s. | n.s. | n.s. | n.s. |
| C - O | n.s. | n.s. | n.s. | n.s. | n.s. | n.s. | n.s. | n.s. | n.s. |
| F - O | n.s. | n.s. | n.s. | n.s. | n.s. | n.s. | n.s. | n.s. | n.s. |
| P - T | n.s. | n.s. | n.s. | n.s. | n.s. | n.s. | n.s. | n.s. | n.s. |
| C - T | n.s. | n.s. | n.s. | n.s. | n.s. | n.s. | n.s. | n.s. | n.s. |
| F - T | n.s. | n.s. | n.s. | n.s. | n.s. | n.s. | n.s. | n.s. | n.s. |
| C - P | n.s. | n.s. | n.s. | n.s. | n.s. | n.s. | n.s. | n.s. | n.s. |
| F - P | n.s. | n.s. | n.s. | n.s. | n.s. | n.s. | n.s. | n.s. | n.s. |
| C - F | n.s. | n.s. | n.s. | n.s. | n.s. | n.s. | n.s. | n.s. | n.s. |
| Age |  |  |  |  |  |  |  |  |  |
| T - O | n.s. | n.s. | n.s. | n.s. | n.s. | n.s. | n.s. | n.s. | n.s. |
| P - O | n.s. | n.s. | n.s. | -.320 | n.s. | n.s. | n.s. | n.s. | n.s. |
| C - O | n.s. | n.s. | n.s. | n.s. | n.s. | n.s. | n.s. | n.s. | n.s. |
| F - O | n.s. | n.s. | n.s. | n.s. | n.s. | n.s. | n.s. | n.s. | n.s. |
| P - T | n.s. | n.s. | n.s. | n.s. | n.s. | n.s. | n.s. | n.s. | .404 |
| C - T | n.s. | n.s. | n.s. | .337 | .306 | n.s. | n.s. | n.s. | .379 |
| F - T | n.s. | n.s. | n.s. | n.s. | .302 | n.s. | n.s. | n.s. | n.s. |
| C - P | n.s. | n.s. | n.s. | n.s. | n.s. | n.s. | n.s. | n.s. | .414 |
| F - P | n.s. | n.s. | n.s. | n.s. | n.s. | n.s. | n.s. | n.s. | n.s. |
| C - F | n.s. | n.s. | n.s. | n.s. | .349 | n.s. | n.s. | n.s. | n.s. |

F=frontal; C=central; P=parietal; O=occipital; T=temporal; n.s.=not significant. The standardised regression coefficient β values are presented for P<0.05.

There was no significance for any interhemispheric coherence at an alpha level of 0.00056. Please note that significance at an alpha level of 0.05 involves the risk of a Type I error.

Table S2. Correlations between coherences in right hemisphere and pattern reasoning ability calculated for all children (*n*=52, ASD and TD children). The standardised regression coefficient β values for the multiple regression model are shown with intrahemispheric coherences in right hemisphere. The Matrix Analogies subtest of the K-ABC and age were applied as independent variables.

| Matrix Analogies | Delta | Theta-1 | Theta-2 | Alpha-1 | Alpha-2 | Beta-1 | Beta-2 | Gamma-1 | Gamma-2 |
| --- | --- | --- | --- | --- | --- | --- | --- | --- | --- |
| T - O | n.s. | n.s. | n.s. | n.s. | n.s. | n.s. | n.s. | n.s. | n.s. |
| P - O | n.s. | n.s. | n.s. | n.s. | n.s. | n.s. | n.s. | n.s. | n.s. |
| C - O | n.s. | n.s. | n.s. | n.s. | n.s. | n.s. | n.s. | n.s. | n.s. |
| F - O | n.s. | n.s. | n.s. | n.s. | n.s. | n.s. | n.s. | n.s. | n.s. |
| P - T | n.s. | n.s. | n.s. | n.s. | n.s. | n.s. | .362 | n.s. | .368 |
| C - T | n.s. | n.s. | n.s. | n.s. | n.s. | n.s. | n.s. | n.s. | n.s. |
| F - T | n.s. | n.s. | n.s. | n.s. | n.s. | n.s. | n.s. | n.s. | n.s. |
| C - P | n.s. | n.s. | n.s. | n.s. | n.s. | n.s. | n.s. | n.s. | n.s. |
| F - P | n.s. | .360 | n.s. | n.s. | n.s. | n.s. | n.s. | n.s. | n.s. |
| C - F | n.s. | n.s. | n.s. | n.s. | n.s. | n.s. | n.s. | n.s. | n.s. |
| Age |  |  |  |  |  |  |  |  |  |
| T - O | n.s. | n.s. | n.s. | n.s. | n.s. | n.s. | n.s. | n.s. | n.s. |
| P - O | n.s. | n.s. | n.s. | n.s. | n.s. | n.s. | n.s. | n.s. | n.s. |
| C - O | n.s. | n.s. | n.s. | n.s. | n.s. | n.s. | n.s. | n.s. | n.s. |
| F - O | n.s. | n.s. | n.s. | n.s. | n.s. | n.s. | n.s. | n.s. | n.s. |
| P - T | n.s. | n.s. | n.s. | n.s. | n.s. | n.s. | n.s. | n.s. | n.s. |
| C - T | n.s. | n.s. | n.s. | n.s. | n.s. | n.s. | n.s. | n.s. | n.s. |
| F - T | n.s. | n.s. | n.s. | n.s. | n.s. | n.s. | n.s. | n.s. | n.s. |
| C - P | n.s. | n.s. | n.s. | n.s. | n.s. | n.s. | n.s. | n.s. | n.s. |
| F - P | n.s. | n.s. | n.s. | n.s. | n.s. | n.s. | n.s. | n.s. | n.s. |
| C - F | n.s. | n.s. | n.s. | n.s. | n.s. | n.s. | n.s. | n.s. | n.s. |

F=frontal; C=central; P=parietal; O=occipital; T=temporal; n.s.=not significant. The standardised regression coefficient β values are presented for P<0.05.

There was no significance for any interhemispheric coherence at an alpha level of 0.00056. Please note that significance at an alpha level of 0.05 involves the risk of a Type I error.

Table S3. Correlations between laterality indices (LIs) of coherences and pattern reasoning ability calculated for all children (*n*=52, ASD and TD children).The standardised regression coefficient β values for the multiple regression model are shown with the LIs calculated for each intrahemispheric coherence. The Matrix Analogies subtest of the K-ABC and age were applied as independent variables.

| Matrix Analogies | Delta | Theta-1 | Theta-2 | Alpha-1 | Alpha-2 | Beta-1 | Beta-2 | Gamma-1 | Gamma-2 |
| --- | --- | --- | --- | --- | --- | --- | --- | --- | --- |
| T - O | n.s. | n.s. | n.s. | n.s. | n.s. | n.s. | n.s. | n.s. | n.s. |
| P - O | n.s. | n.s. | n.s. | n.s. | n.s. | n.s. | n.s. | n.s. | n.s. |
| C - O | n.s. | n.s. | n.s. | n.s. | n.s. | n.s. | n.s. | -.308 | n.s. |
| F - O | n.s. | n.s. | n.s. | n.s. | n.s. | n.s. | n.s. | n.s. | n.s. |
| P - T | n.s. | n.s. | n.s. | n.s. | n.s. | n.s. | n.s. | n.s. | -.371 |
| C - T | n.s. | n.s. | n.s. | n.s. | n.s. | n.s. | n.s. | n.s. | n.s. |
| F - T | n.s. | n.s. | n.s. | -.331 | n.s. | n.s. | n.s. | n.s. | n.s. |
| C - P | n.s. | n.s. | n.s. | n.s. | n.s. | n.s. | n.s. | n.s. | n.s. |
| F - P | n.s. | n.s. | n.s. | n.s. | n.s. | n.s. | n.s. | n.s. | n.s. |
| C - F | n.s. | n.s. | n.s. | n.s. | n.s. | n.s. | n.s. | -.312 | n.s. |
| Age |  |  |  |  |  |  |  |  |  |
| T - O | n.s. | n.s. | n.s. | n.s. | n.s. | n.s. | n.s. | n.s. | n.s. |
| P - O | n.s. | n.s. | n.s. | n.s. | n.s. | n.s. | n.s. | n.s. | n.s. |
| C - O | n.s. | n.s. | n.s. | n.s. | n.s. | n.s. | n.s. | n.s. | n.s. |
| F - O | n.s. | .346 | n.s. | n.s. | n.s. | n.s. | n.s. | n.s. | n.s. |
| P - T | n.s. | n.s. | n.s. | n.s. | n.s. | n.s. | n.s. | n.s. | .491 |
| C - T | n.s. | n.s. | n.s. | n.s. | n.s. | n.s. | n.s. | n.s. | .445 |
| F - T | n.s. | n.s. | n.s. | n.s. | n.s. | n.s. | n.s. | n.s. | n.s. |
| C - P | n.s. | n.s. | n.s. | n.s. | n.s. | n.s. | n.s. | n.s. | .381 |
| F - P | n.s. | n.s. | n.s. | n.s. | n.s. | n.s. | n.s. | n.s. | n.s. |
| C - F | n.s. | n.s. | n.s. | n.s. | .415 | n.s. | n.s. | n.s. | n.s. |

F=frontal; C=central; P=parietal; O=occipital; T=temporal; n.s.=not significant. The standardised regression coefficient β values are presented for P<0.05.

There was no significance for any LI at an alpha level of 0.00056. Please note that significance at an alpha level of 0.05 involves the risk of a Type I error.

Table S4. Correlations between coherences in left hemisphere and pattern reasoning ability calculated for children with ASD (*n*=26). The standardised regression coefficient β values for the multiple regression model are shown with intrahemispheric coherences in left hemisphere. The Matrix Analogies subtest of the K-ABC and age were applied as independent variables.

| Matrix Analogies | Delta | Theta-1 | Theta-2 | Alpha-1 | Alpha-2 | Beta-1 | Beta-2 | Gamma-1 | Gamma-2 |
| --- | --- | --- | --- | --- | --- | --- | --- | --- | --- |
| T - O | n.s. | n.s. | n.s. | n.s. | n.s. | n.s. | n.s. | n.s. | n.s. |
| P - O | n.s. | n.s. | n.s. | n.s. | n.s. | n.s. | n.s. | n.s. | n.s. |
| C - O | n.s. | n.s. | n.s. | n.s. | n.s. | n.s. | n.s. | -.450 | n.s. |
| F - O | n.s. | n.s. | n.s. | n.s. | n.s. | n.s. | n.s. | n.s. | n.s. |
| P - T | n.s. | n.s. | n.s. | n.s. | n.s. | n.s. | n.s. | n.s. | n.s. |
| C - T | n.s. | n.s. | n.s. | n.s. | n.s. | n.s. | n.s. | n.s. | n.s. |
| F - T | n.s. | n.s. | n.s. | n.s. | n.s. | n.s. | n.s. | n.s. | n.s. |
| C - P | n.s. | n.s. | n.s. | n.s. | n.s. | n.s. | n.s. | n.s. | n.s. |
| F - P | n.s. | n.s. | n.s. | n.s. | n.s. | n.s. | n.s. | n.s. | n.s. |
| C - F | n.s. | n.s. | n.s. | n.s. | n.s. | n.s. | n.s. | n.s. | n.s. |
| Age |  |  |  |  |  |  |  |  |  |
| T - O | n.s. | n.s. | n.s. | n.s. | n.s. | n.s. | n.s. | n.s. | n.s. |
| P - O | n.s. | n.s. | n.s. | n.s. | n.s. | n.s. | n.s. | n.s. | n.s. |
| C - O | n.s. | n.s. | n.s. | n.s. | n.s. | n.s. | n.s. | n.s. | n.s. |
| F - O | n.s. | n.s. | n.s. | n.s. | n.s. | n.s. | n.s. | n.s. | n.s. |
| P - T | n.s. | n.s. | n.s. | n.s. | n.s. | n.s. | n.s. | n.s. | .559 |
| C - T | n.s. | n.s. | n.s. | n.s. | n.s. | n.s. | n.s. | n.s. | .436 |
| F - T | n.s. | n.s. | n.s. | n.s. | n.s. | n.s. | n.s. | n.s. | n.s. |
| C - P | n.s. | n.s. | n.s. | n.s. | n.s. | n.s. | n.s. | n.s. | .437 |
| F - P | n.s. | n.s. | n.s. | n.s. | .485 | n.s. | n.s. | n.s. | n.s. |
| C - F | n.s. | n.s. | n.s. | n.s. | n.s. | n.s. | n.s. | n.s. | n.s. |

F=frontal; C=central; P=parietal; O=occipital; T=temporal; n.s.=not significant. The standardised regression coefficient β values are presented for P<0.05.

There was no significance for any interhemispheric coherence at an alpha level of 0.00056. Please note that significance at an alpha level of 0.05 involves the risk of a Type I error.

Table S5. Correlations between coherences in right hemisphere and pattern reasoning ability calculated for children with ASD (*n*=26). The standardised regression coefficient β values for the multiple regression model are shown with intrahemispheric coherences in right hemisphere. The Matrix Analogies subtest of the K-ABC and age were applied as independent variables.

| Matrix Analogies | Delta | Theta-1 | Theta-2 | Alpha-1 | Alpha-2 | Beta-1 | Beta-2 | Gamma-1 | Gamma-2 |
| --- | --- | --- | --- | --- | --- | --- | --- | --- | --- |
| T - O | n.s. | n.s. | n.s. | n.s. | n.s. | .415 | .489 | n.s. | n.s. |
| P - O | n.s. | n.s. | n.s. | n.s. | n.s. | n.s. | n.s. | n.s. | n.s. |
| C - O | n.s. | n.s. | n.s. | n.s. | n.s. | n.s. | n.s. | n.s. | n.s. |
| F - O | n.s. | n.s. | n.s. | n.s. | n.s. | n.s. | n.s. | n.s. | n.s. |
| P - T | n.s. | .419 | n.s. | n.s. | n.s. | n.s. | .510 | .434 | .497 |
| C - T | n.s. | n.s. | n.s. | n.s. | n.s. | n.s. | n.s. | n.s. | n.s. |
| F - T | n.s. | n.s. | n.s. | n.s. | n.s. | n.s. | .404 | n.s. | n.s. |
| C - P | n.s. | n.s. | n.s. | n.s. | n.s. | n.s. | n.s. | n.s. | n.s. |
| F - P | n.s. | n.s. | n.s. | n.s. | n.s. | n.s. | n.s. | n.s. | n.s. |
| C - F | n.s. | n.s. | n.s. | n.s. | n.s. | n.s. | n.s. | n.s. | n.s. |
| Age |  |  |  |  |  |  |  |  |  |
| T - O | n.s. | n.s. | n.s. | n.s. | n.s. | -.489 | .445 | n.s. | n.s. |
| P - O | n.s. | n.s. | n.s. | n.s. | n.s. | n.s. | n.s. | n.s. | n.s. |
| C - O | n.s. | n.s. | n.s. | n.s. | n.s. | n.s. | n.s. | n.s. | n.s. |
| F - O | n.s. | n.s. | n.s. | n.s. | n.s. | n.s. | n.s. | n.s. | n.s. |
| P - T | n.s. | -.445 | n.s. | n.s. | n.s. | n.s. | n.s. | n.s. | n.s. |
| C - T | n.s. | n.s. | n.s. | n.s. | n.s. | n.s. | n.s. | n.s. | n.s. |
| F - T | n.s. | n.s. | n.s. | n.s. | n.s. | n.s. | -.527 | n.s. | n.s. |
| C - P | n.s. | n.s. | n.s. | n.s. | n.s. | n.s. | n.s. | n.s. | n.s. |
| F - P | n.s. | n.s. | n.s. | n.s. | n.s. | n.s. | n.s. | n.s. | n.s. |
| C - F | n.s. | n.s. | n.s. | n.s. | n.s. | n.s. | n.s. | n.s. | n.s. |

F=frontal; C=central; P=parietal; O=occipital; T=temporal; n.s.=not significant. The standardised regression coefficient β values are presented for P<0.05.

There was no significance for any interhemispheric coherence at an alpha level of 0.00056. Please note that significance at an alpha level of 0.05 involves the risk of a Type I error.

Table S6. Correlations between laterality indices (LIs) of coherences and pattern reasoning ability calculated for children with ASD (*n*=26). The standardised regression coefficient β values for the multiple regression model are shown with the LIs calculated for each intrahemispheric coherence. The Matrix Analogies subtest of the K-ABC and age were applied as independent variables.

| Matrix Analogies | Delta | Theta-1 | Theta-2 | Alpha-1 | Alpha-2 | Beta-1 | Beta-2 | Gamma-1 | Gamma-2 |
| --- | --- | --- | --- | --- | --- | --- | --- | --- | --- |
| T - O | n.s. | n.s. | n.s. | n.s. | n.s. | n.s. | n.s. | n.s. | n.s. |
| P - O | n.s. | n.s. | n.s. | n.s. | n.s. | n.s. | .497 | n.s. | n.s. |
| C - O | n.s. | n.s. | n.s. | n.s. | n.s. | n.s. | n.s. | n.s. | n.s. |
| F - O | n.s. | n.s. | n.s. | n.s. | n.s. | n.s. | n.s. | n.s. | n.s. |
| P - T | n.s. | n.s. | n.s. | n.s. | n.s. | n.s. | n.s. | -.491 | -.641* |
| C - T | n.s. | n.s. | n.s. | n.s. | n.s. | n.s. | n.s. | -.443 | n.s. |
| F - T | n.s. | n.s. | n.s. | -.453 | n.s. | n.s. | n.s. | n.s. | n.s. |
| C - P | n.s. | n.s. | -.555 | n.s. | n.s. | n.s. | n.s. | n.s. | n.s. |
| F - P | n.s. | n.s. | n.s. | n.s. | n.s. | n.s. | n.s. | n.s. | n.s. |
| C - F | n.s. | n.s. | n.s. | n.s. | -.556 | n.s. | n.s. | -.482 | n.s. |
| Age |  |  |  |  |  |  |  |  |  |
| T - O | n.s. | n.s. | n.s. | n.s. | n.s. | n.s. | n.s. | n.s. | n.s. |
| P - O | n.s. | n.s. | n.s. | n.s. | n.s. | n.s. | n.s. | n.s. | n.s. |
| C - O | n.s. | n.s. | n.s. | n.s. | n.s. | n.s. | n.s. | n.s. | n.s. |
| F - O | n.s. | n.s. | n.s. | n.s. | n.s. | n.s. | n.s. | n.s. | n.s. |
| P - T | n.s. | n.s. | n.s. | n.s. | n.s. | n.s. | n.s. | n.s. | .682* |
| C - T | n.s. | n.s. | n.s. | n.s. | n.s. | n.s. | n.s. | n.s. | .573 |
| F - T | n.s. | n.s. | .433 | n.s. | n.s. | n.s. | .436 | n.s. | n.s. |
| C - P | n.s. | n.s. | .433 | n.s. | n.s. | n.s. | n.s. | n.s. | .487 |
| F - P | n.s. | n.s. | n.s. | -.457 | n.s. | n.s. | n.s. | n.s. | n.s. |
| C - F | n.s. | n.s. | n.s. | n.s. | .617 | n.s. | n.s. | n.s. | n.s. |

F=frontal; C=central; P=parietal; O=occipital; T=temporal; n.s.=not significant. The standardised regression coefficient β values are presented for P<0.05.

**P*<0.00056. Please note that significance at an alpha level of 0.05 involves the risk of a Type I error.

Table S7. Correlations between coherences in left hemisphere and pattern reasoning ability calculated for TD children (*n*=26). The standardised regression coefficient β values for the multiple regression model are shown with intrahemispheric coherences in left hemisphere. The Matrix Analogies subtest of the K-ABC and age were applied as independent variables.

| Matrix Analogies | Delta | Theta-1 | Theta-2 | Alpha-1 | Alpha-2 | Beta-1 | Beta-2 | Gamma-1 | Gamma-2 |
| --- | --- | --- | --- | --- | --- | --- | --- | --- | --- |
| T - O | n.s. | n.s. | n.s. | n.s. | n.s. | n.s. | n.s. | n.s. | n.s. |
| P - O | n.s. | n.s. | n.s. | n.s. | n.s. | n.s. | n.s. | n.s. | n.s. |
| C - O | n.s. | n.s. | n.s. | n.s. | n.s. | n.s. | n.s. | n.s. | n.s. |
| F - O | n.s. | n.s. | n.s. | n.s. | n.s. | n.s. | n.s. | n.s. | n.s. |
| P - T | n.s. | n.s. | n.s. | n.s. | .438 | n.s. | n.s. | n.s. | n.s. |
| C - T | n.s. | n.s. | n.s. | n.s. | n.s. | n.s. | n.s. | n.s. | n.s. |
| F - T | n.s. | n.s. | n.s. | n.s. | n.s. | n.s. | n.s. | n.s. | n.s. |
| C - P | n.s. | n.s. | n.s. | n.s. | n.s. | n.s. | n.s. | n.s. | n.s. |
| F - P | n.s. | n.s. | n.s. | n.s. | n.s. | n.s. | n.s. | n.s. | n.s. |
| C - F | n.s. | n.s. | n.s. | n.s. | .449 | n.s. | n.s. | n.s. | n.s. |
| Age |  |  |  |  |  |  |  |  |  |
| T - O | n.s. | n.s. | n.s. | n.s. | n.s. | n.s. | n.s. | n.s. | n.s. |
| P - O | n.s. | n.s. | n.s. | n.s. | n.s. | n.s. | n.s. | n.s. | n.s. |
| C - O | n.s. | n.s. | n.s. | n.s. | n.s. | n.s. | .563 | n.s. | n.s. |
| F - O | n.s. | n.s. | n.s. | n.s. | n.s. | n.s. | n.s. | n.s. | n.s. |
| P - T | n.s. | n.s. | n.s. | n.s. | n.s. | n.s. | n.s. | n.s. | n.s. |
| C - T | n.s. | n.s. | n.s. | n.s. | n.s. | n.s. | .552 | n.s. | n.s. |
| F - T | n.s. | n.s. | n.s. | n.s. | n.s. | n.s. | n.s. | n.s. | n.s. |
| C - P | n.s. | n.s. | n.s. | n.s. | n.s. | n.s. | n.s. | n.s. | n.s. |
| F - P | n.s. | n.s. | n.s. | n.s. | n.s. | n.s. | n.s. | n.s. | n.s. |
| C - F | n.s. | n.s. | n.s. | n.s. | n.s. | n.s. | n.s. | n.s. | n.s. |

F=frontal; C=central; P=parietal; O=occipital; T=temporal; n.s.=not significant. The standardised regression coefficient β values are presented for P<0.05.

There was no significance for any interhemispheric coherence at an alpha level of 0.00056. Please note that significance at an alpha level of 0.05 involves the risk of a Type I error.

Table S8. Correlations between coherences in right hemisphere and pattern reasoning ability calculated for TD children (*n*=26). The standardised regression coefficient β values for the multiple regression model are shown with intrahemispheric coherences in right hemisphere. The Matrix Analogies subtest of the K-ABC and age were applied as independent variables.

| Matrix Analogies | Delta | Theta-1 | Theta-2 | Alpha-1 | Alpha-2 | Beta-1 | Beta-2 | Gamma-1 | Gamma-2 |
| --- | --- | --- | --- | --- | --- | --- | --- | --- | --- |
| T - O | n.s. | n.s. | n.s. | n.s. | n.s. | n.s. | -.481 | n.s. | n.s. |
| P - O | n.s. | n.s. | n.s. | n.s. | n.s. | n.s. | n.s. | n.s. | n.s. |
| C - O | n.s. | n.s. | n.s. | n.s. | n.s. | n.s. | n.s. | n.s. | n.s. |
| F - O | n.s. | n.s. | .501 | n.s. | n.s. | n.s. | n.s. | n.s. | n.s. |
| P - T | n.s. | n.s. | n.s. | n.s. | n.s. | n.s. | n.s. | n.s. | n.s. |
| C - T | n.s. | n.s. | n.s. | n.s. | n.s. | n.s. | n.s. | n.s. | n.s. |
| F - T | n.s. | n.s. | n.s. | n.s. | n.s. | n.s. | n.s. | n.s. | n.s. |
| C - P | n.s. | n.s. | n.s. | n.s. | n.s. | n.s. | n.s. | n.s. | n.s. |
| F - P | n.s. | n.s. | n.s. | n.s. | n.s. | n.s. | n.s. | n.s. | n.s. |
| C - F | n.s. | n.s. | n.s. | n.s. | n.s. | n.s. | n.s. | n.s. | n.s. |
| Age |  |  |  |  |  |  |  |  |  |
| T - O | n.s. | n.s. | n.s. | n.s. | n.s. | n.s. | n.s. | n.s. | n.s. |
| P - O | n.s. | n.s. | n.s. | n.s. | n.s. | n.s. | n.s. | n.s. | n.s. |
| C - O | n.s. | n.s. | n.s. | n.s. | n.s. | n.s. | n.s. | n.s. | n.s. |
| F - O | n.s. | n.s. | n.s. | n.s. | n.s. | n.s. | n.s. | n.s. | n.s. |
| P - T | n.s. | n.s. | n.s. | n.s. | n.s. | n.s. | n.s. | n.s. | n.s. |
| C - T | n.s. | n.s. | n.s. | n.s. | n.s. | n.s. | n.s. | n.s. | n.s. |
| F - T | n.s. | n.s. | n.s. | n.s. | n.s. | .431 | .461 | n.s. | n.s. |
| C - P | n.s. | n.s. | n.s. | n.s. | n.s. | n.s. | n.s. | n.s. | n.s. |
| F - P | n.s. | n.s. | n.s. | n.s. | n.s. | n.s. | n.s. | n.s. | n.s. |
| C - F | n.s. | n.s. | n.s. | n.s. | n.s. | n.s. | n.s. | n.s. | n.s. |

F=frontal; C=central; P=parietal; O=occipital; T=temporal; n.s.=not significant. The standardised regression coefficient β values are presented for P<0.05.

There was no significance for any interhemispheric coherence at an alpha level of 0.00056. Please note that significance at an alpha level of 0.05 involves the risk of a Type I error.

Table S9. Correlations between laterality indices (LIs) of coherences and pattern reasoning ability calculated for TD children (*n*=26).The standardised regression coefficient β values for the multiple regression model are shown with the LIs calculated for each intrahemispheric coherence. The Matrix Analogies subtest of the K-ABC and age were applied as independent variables.

(b) TD children

| Matrix Analogies | Delta | Theta-1 | Theta-2 | Alpha-1 | Alpha-2 | Beta-1 | Beta-2 | Gamma-1 | Gamma-2 |
| --- | --- | --- | --- | --- | --- | --- | --- | --- | --- |
| T - O | n.s. | n.s. | n.s. | n.s. | n.s. | .573 | n.s. | n.s. | n.s. |
| P - O | n.s. | n.s. | n.s. | n.s. | n.s. | n.s. | n.s. | n.s. | n.s. |
| C - O | n.s. | n.s. | n.s. | n.s. | n.s. | n.s. | n.s. | n.s. | n.s. |
| F - O | n.s. | n.s. | n.s. | n.s. | n.s. | n.s. | n.s. | n.s. | n.s. |
| P - T | n.s. | n.s. | n.s. | n.s. | .503 | n.s. | n.s. | .492 | n.s. |
| C - T | n.s. | n.s. | n.s. | n.s. | n.s. | n.s. | n.s. | n.s. | n.s. |
| F - T | n.s. | n.s. | n.s. | n.s. | n.s. | n.s. | n.s. | n.s. | n.s. |
| C - P | n.s. | n.s. | n.s. | .563 | n.s. | n.s. | n.s. | n.s. | n.s. |
| F - P | n.s. | n.s. | n.s. | n.s. | .450 | n.s. | n.s. | n.s. | n.s. |
| C - F | n.s. | n.s. | n.s. | n.s. | n.s. | n.s. | n.s. | n.s. | n.s. |
| Age |  |  |  |  |  |  |  |  |  |
| T - O | n.s. | n.s. | n.s. | n.s. | n.s. | -.546 | n.s. | n.s. | n.s. |
| P - O | n.s. | n.s. | n.s. | n.s. | n.s. | n.s. | n.s. | n.s. | n.s. |
| C - O | n.s. | n.s. | n.s. | n.s. | n.s. | n.s. | n.s. | n.s. | n.s. |
| F - O | n.s. | .488 | n.s. | n.s. | n.s. | n.s. | n.s. | n.s. | n.s. |
| P - T | n.s. | n.s. | n.s. | n.s. | n.s. | n.s. | n.s. | n.s. | n.s. |
| C - T | n.s. | n.s. | n.s. | n.s. | n.s. | n.s. | .544 | n.s. | n.s. |
| F - T | n.s. | n.s. | n.s. | n.s. | n.s. | n.s. | -.447 | n.s. | n.s. |
| C - P | n.s. | n.s. | n.s. | -.614 | n.s. | n.s. | n.s. | n.s. | n.s. |
| F - P | n.s. | n.s. | n.s. | n.s. | n.s. | n.s. | n.s. | n.s. | n.s. |
| C - F | n.s. | n.s. | n.s. | n.s. | n.s. | n.s. | n.s. | n.s. | n.s. |

F=frontal; C=central; P=parietal; O=occipital; T=temporal; n.s.=not significant. The standardised regression coefficient β values are presented for P<0.05.

There was no significance for any LI at an alpha level of 0.00056. Please note that significance at an alpha level of 0.05 involves the risk of a Type I error.

Table S10. Correlations between coherences in left hemisphere and reading ability calculated for all children (*n*=52, ASD and TD children). The standardised regression coefficient β values for the multiple regression model are shown with intrahemispheric coherences in left hemisphere. The K-ABC subtest “Reading/Decoding” and age were applied as independent variables.

| Reading/  Decoding | Delta | Theta-1 | Theta-2 | Alpha-1 | Alpha-2 | Beta-1 | Beta-2 | Gamma-1 | Gamma-2 |
| --- | --- | --- | --- | --- | --- | --- | --- | --- | --- |
| T - O | n.s. | n.s. | n.s. | n.s. | n.s. | n.s. | n.s. | n.s. | n.s. |
| P - O | n.s. | n.s. | n.s. | n.s. | n.s. | n.s. | n.s. | n.s. | n.s. |
| C - O | n.s. | n.s. | n.s. | n.s. | n.s. | n.s. | n.s. | n.s. | n.s. |
| F - O | n.s. | n.s. | n.s. | n.s. | n.s. | n.s. | n.s. | n.s. | n.s. |
| P - T | n.s. | n.s. | n.s. | n.s. | n.s. | n.s. | n.s. | n.s. | n.s. |
| C - T | n.s. | n.s. | n.s. | n.s. | .505 | n.s. | .432 | n.s. | n.s. |
| F - T | n.s. | n.s. | n.s. | n.s. | n.s. | n.s. | .503 | n.s. | n.s. |
| C - P | n.s. | n.s. | n.s. | n.s. | n.s. | n.s. | n.s. | n.s. | n.s. |
| F - P | n.s. | n.s. | n.s. | n.s. | .466 | n.s. | n.s. | n.s. | n.s. |
| C - F | n.s. | n.s. | n.s. | n.s. | .311 | n.s. | n.s. | n.s. | n.s. |
| Age |  |  |  |  |  |  |  |  |  |
| T - O | n.s. | n.s. | n.s. | n.s. | n.s. | n.s. | n.s. | n.s. | n.s. |
| P - O | n.s. | n.s. | n.s. | -.380 | n.s. | n.s. | n.s. | n.s. | n.s. |
| C - O | n.s. | n.s. | n.s. | n.s. | n.s. | n.s. | n.s. | n.s. | n.s. |
| F - O | n.s. | n.s. | n.s. | n.s. | n.s. | n.s. | n.s. | n.s. | n.s. |
| P - T | n.s. | n.s. | n.s. | n.s. | n.s. | n.s. | n.s. | n.s. | n.s. |
| C - T | n.s. | n.s. | n.s. | n.s. | n.s. | n.s. | n.s. | n.s. | .387 |
| F - T | n.s. | n.s. | n.s. | n.s. | n.s. | n.s. | n.s. | n.s. | n.s. |
| C - P | n.s. | n.s. | n.s. | n.s. | n.s. | n.s. | n.s. | n.s. | .362 |
| F - P | n.s. | n.s. | n.s. | n.s. | n.s. | n.s. | n.s. | n.s. | n.s. |
| C - F | n.s. | n.s. | n.s. | n.s. | n.s. | n.s. | n.s. | n.s. | n.s. |

F=frontal; C=central; P=parietal; O=occipital; T=temporal; n.s.=not significant. The standardised regression coefficient β values are presented for P<0.05.

There was no significance for any interhemispheric coherence at an alpha level of 0.00056. Please note that significance at an alpha level of 0.05 involves the risk of a Type I error.

Table S11. Correlations between coherences in right hemisphere and reading ability calculated for all children (*n*=52, ASD and TD children). The standardised regression coefficient β values for the multiple regression model are shown with intrahemispheric coherences in right hemisphere. The K-ABC subtest “Reading/Decoding” and age were applied as independent variables.

| Reading/  Decoding | Delta | Theta-1 | Theta-2 | Alpha-1 | Alpha-2 | Beta-1 | Beta-2 | Gamma-1 | Gamma-2 |
| --- | --- | --- | --- | --- | --- | --- | --- | --- | --- |
| T - O | n.s. | n.s. | n.s. | n.s. | n.s. | n.s. | n.s. | n.s. | n.s. |
| P - O | n.s. | n.s. | n.s. | n.s. | n.s. | n.s. | n.s. | n.s. | n.s. |
| C - O | n.s. | n.s. | n.s. | n.s. | n.s. | n.s. | n.s. | n.s. | n.s. |
| F - O | n.s. | n.s. | n.s. | n.s. | n.s. | n.s. | n.s. | n.s. | n.s. |
| P - T | n.s. | n.s. | n.s. | n.s. | n.s. | n.s. | n.s. | .489 | .546 |
| C - T | n.s. | n.s. | n.s. | n.s. | n.s. | n.s. | n.s. | n.s. | n.s. |
| F - T | n.s. | n.s. | n.s. | n.s. | n.s. | n.s. | n.s. | n.s. | n.s. |
| C - P | n.s. | n.s. | n.s. | n.s. | .341 | n.s. | n.s. | n.s. | n.s. |
| F - P | n.s. | n.s. | n.s. | n.s. | n.s. | n.s. | n.s. | n.s. | n.s. |
| C - F | n.s. | n.s. | n.s. | n.s. | n.s. | n.s. | n.s. | n.s. | n.s. |
| Age |  |  |  |  |  |  |  |  |  |
| T - O | n.s. | n.s. | n.s. | n.s. | n.s. | n.s. | n.s. | n.s. | n.s. |
| P - O | n.s. | n.s. | n.s. | n.s. | n.s. | n.s. | n.s. | n.s. | n.s. |
| C - O | n.s. | n.s. | n.s. | n.s. | n.s. | n.s. | n.s. | n.s. | n.s. |
| F - O | n.s. | n.s. | n.s. | n.s. | n.s. | n.s. | n.s. | n.s. | n.s. |
| P - T | n.s. | n.s. | n.s. | n.s. | n.s. | n.s. | .491 | n.s. | n.s. |
| C - T | n.s. | n.s. | n.s. | n.s. | n.s. | n.s. | n.s. | n.s. | n.s. |
| F - T | n.s. | n.s. | n.s. | n.s. | n.s. | n.s. | n.s. | n.s. | n.s. |
| C - P | n.s. | n.s. | n.s. | n.s. | n.s. | n.s. | n.s. | n.s. | n.s. |
| F - P | n.s. | n.s. | n.s. | n.s. | n.s. | n.s. | n.s. | n.s. | n.s. |
| C - F | n.s. | n.s. | n.s. | n.s. | n.s. | n.s. | n.s. | n.s. | n.s. |

F=frontal; C=central; P=parietal; O=occipital; T=temporal; n.s.=not significant. The standardised regression coefficient β values are presented for P<0.05.

There was no significance for any interhemispheric coherence at an alpha level of 0.00056. Please note that significance at an alpha level of 0.05 involves the risk of a Type I error.

Table S12. Correlations between laterality indices (LIs) of coherences and reading ability calculated for all children (*n*=52, ASD and TD children).The standardised regression coefficient β values for the multiple regression model are shown with the LIs calculated for each intrahemispheric coherence. The K-ABC subtest “Reading/Decoding” and age were applied as independent variables.

| Reading/  Decoding | Delta | Theta-1 | Theta-2 | Alpha-1 | Alpha-2 | Beta-1 | Beta-2 | Gamma-1 | Gamma-2 |
| --- | --- | --- | --- | --- | --- | --- | --- | --- | --- |
| T - O | n.s. | n.s. | n.s. | n.s. | n.s. | n.s. | n.s. | n.s. | n.s. |
| P - O | n.s. | -.372 | n.s. | n.s. | n.s. | n.s. | n.s. | n.s. | n.s. |
| C - O | n.s. | n.s. | n.s. | n.s. | n.s. | n.s. | n.s. | n.s. | n.s. |
| F - O | n.s. | n.s. | n.s. | n.s. | n.s. | n.s. | -.370 | n.s. | n.s. |
| P - T | n.s. | n.s. | n.s. | n.s. | n.s. | n.s. | n.s. | n.s. | -.437 |
| C - T | n.s. | n.s. | n.s. | n.s. | n.s. | n.s. | n.s. | n.s. | n.s. |
| F - T | -.347 | n.s. | n.s. | n.s. | .447 | n.s. | .336 | n.s. | n.s. |
| C - P | n.s. | n.s. | n.s. | n.s. | n.s. | n.s. | n.s. | n.s. | n.s. |
| F - P | n.s. | n.s. | n.s. | n.s. | n.s. | n.s. | n.s. | n.s. | n.s. |
| C - F | n.s. | n.s. | n.s. | n.s. | n.s. | n.s. | n.s. | n.s. | n.s. |
| Age |  |  |  |  |  |  |  |  |  |
| T - O | n.s. | n.s. | n.s. | n.s. | -.338 | n.s. | n.s. | n.s. | n.s. |
| P - O | n.s. | n.s. | n.s. | n.s. | n.s. | n.s. | n.s. | n.s. | n.s. |
| C - O | n.s. | n.s. | n.s. | n.s. | n.s. | n.s. | n.s. | n.s. | n.s. |
| F - O | n.s. | .393 | n.s. | n.s. | n.s. | n.s. | n.s. | n.s. | n.s. |
| P - T | n.s. | n.s. | n.s. | n.s. | n.s. | n.s. | n.s. | n.s. | .574 * |
| C - T | n.s. | n.s. | n.s. | n.s. | n.s. | n.s. | n.s. | n.s. | .466 |
| F - T | n.s. | n.s. | n.s. | n.s. | n.s. | -.339 | n.s. | n.s. | n.s. |
| C - P | n.s. | n.s. | n.s. | n.s. | n.s. | n.s. | n.s. | n.s. | n.s. |
| F - P | n.s. | n.s. | n.s. | n.s. | n.s. | n.s. | n.s. | n.s. | n.s. |
| C - F | n.s. | n.s. | n.s. | n.s. | n.s. | n.s. | n.s. | n.s. | n.s. |

F=frontal; C=central; P=parietal; O=occipital; T=temporal; n.s.=not significant. The standardised regression coefficient β values are presented for P<0.05.

**P*<0.00056. Please note that significance at an alpha level of 0.05 involves the risk of a Type I error.

Table S13. Correlations between coherences in left hemisphere and reading ability calculated for children with ASD (*n*=26). The standardised regression coefficient β values for the multiple regression model are shown with intrahemispheric coherences in left hemisphere. The K-ABC subtest “Reading/Decoding” and age were applied as independent variables.

| Reading/  Decoding | Delta | Theta-1 | Theta-2 | Alpha-1 | Alpha-2 | Beta-1 | Beta-2 | Gamma-1 | Gamma-2 |
| --- | --- | --- | --- | --- | --- | --- | --- | --- | --- |
| T - O | n.s. | n.s. | n.s. | n.s. | n.s. | n.s. | n.s. | n.s. | n.s. |
| P - O | n.s. | n.s. | n.s. | n.s. | n.s. | n.s. | n.s. | n.s. | n.s. |
| C - O | n.s. | n.s. | n.s. | n.s. | n.s. | n.s. | n.s. | n.s. | n.s. |
| F - O | n.s. | n.s. | n.s. | n.s. | n.s. | n.s. | n.s. | n.s. | n.s. |
| P - T | n.s. | n.s. | n.s. | n.s. | n.s. | n.s. | n.s. | .557 | n.s. |
| C - T | n.s. | n.s. | n.s. | n.s. | .618 | n.s. | .596 | n.s. | n.s. |
| F - T | n.s. | n.s. | n.s. | n.s. | n.s. | n.s. | .532 | n.s. | n.s. |
| C - P | n.s. | n.s. | n.s. | n.s. | n.s. | n.s. | n.s. | n.s. | n.s. |
| F - P | n.s. | n.s. | n.s. | .532 | .480 | n.s. | n.s. | .571 | n.s. |
| C - F | n.s. | n.s. | n.s. | n.s. | n.s. | n.s. | n.s. | n.s. | n.s. |
| Age |  |  |  |  |  |  |  |  |  |
| T - O | n.s. | n.s. | n.s. | n.s. | n.s. | n.s. | n.s. | n.s. | n.s. |
| P - O | n.s. | n.s. | n.s. | n.s. | n.s. | n.s. | n.s. | n.s. | n.s. |
| C - O | n.s. | n.s. | n.s. | n.s. | n.s. | n.s. | n.s. | n.s. | n.s. |
| F - O | n.s. | n.s. | n.s. | n.s. | n.s. | n.s. | n.s. | n.s. | n.s. |
| P - T | n.s. | -.588 | n.s. | n.s. | n.s. | n.s. | n.s. | n.s. | n.s. |
| C - T | n.s. | n.s. | n.s. | n.s. | n.s. | n.s. | n.s. | n.s. | n.s. |
| F - T | n.s. | n.s. | n.s. | n.s. | n.s. | n.s. | n.s. | n.s. | n.s. |
| C - P | n.s. | n.s. | n.s. | n.s. | n.s. | n.s. | n.s. | n.s. | n.s. |
| F - P | n.s. | n.s. | n.s. | -.532 | n.s. | n.s. | n.s. | n.s. | n.s. |
| C - F | n.s. | n.s. | n.s. | n.s. | n.s. | n.s. | n.s. | n.s. | n.s. |

F=frontal; C=central; P=parietal; O=occipital; T=temporal; n.s.=not significant. The standardised regression coefficient β values are presented for P<0.05.

There was no significance for any interhemispheric coherence at an alpha level of 0.00056. Please note that significance at an alpha level of 0.05 involves the risk of a Type I error.

Table S14. Correlations between coherences in right hemisphere and reading ability calculated for children with ASD (*n*=26). The standardised regression coefficient β values for the multiple regression model are shown with intrahemispheric coherences in right hemisphere. The K-ABC subtest “Reading/Decoding” and age were applied as independent variables.

| Reading/  Decoding | Delta | Theta-1 | Theta-2 | Alpha-1 | Alpha-2 | Beta-1 | Beta-2 | Gamma-1 | Gamma-2 |
| --- | --- | --- | --- | --- | --- | --- | --- | --- | --- |
| T - O | n.s. | n.s. | n.s. | n.s. | n.s. | n.s. | n.s. | n.s. | n.s. |
| P - O | n.s. | n.s. | n.s. | n.s. | n.s. | n.s. | n.s. | n.s. | n.s. |
| C - O | n.s. | n.s. | n.s. | n.s. | n.s. | n.s. | n.s. | n.s. | n.s. |
| F - O | n.s. | n.s. | n.s. | n.s. | n.s. | n.s. | n.s. | n.s. | n.s. |
| P - T | n.s. | n.s. | n.s. | n.s. | n.s. | n.s. | .794 | .691 | .905* |
| C - T | n.s. | n.s. | n.s. | n.s. | n.s. | n.s. | n.s. | .562 | n.s. |
| F - T | n.s. | n.s. | n.s. | n.s. | n.s. | n.s. | n.s. | n.s. | n.s. |
| C - P | n.s. | n.s. | n.s. | n.s. | n.s. | n.s. | n.s. | n.s. | n.s. |
| F - P | n.s. | n.s. | n.s. | n.s. | n.s. | n.s. | .599 | n.s. | n.s. |
| C - F | n.s. | -.571 | n.s. | n.s. | n.s. | n.s. | n.s. | n.s. | n.s. |
| Age |  |  |  |  |  |  |  |  |  |
| T - O | n.s. | n.s. | n.s. | n.s. | n.s. | -.628 | n.s. | n.s. | n.s. |
| P - O | n.s. | n.s. | n.s. | n.s. | n.s. | n.s. | n.s. | n.s. | n.s. |
| C - O | n.s. | n.s. | n.s. | n.s. | n.s. | n.s. | n.s. | n.s. | n.s. |
| F - O | n.s. | n.s. | n.s. | n.s. | n.s. | n.s. | n.s. | n.s. | n.s. |
| P - T | n.s. | n.s. | n.s. | n.s. | n.s. | n.s. | -.536 | n.s. | -.750 |
| C - T | n.s. | n.s. | n.s. | n.s. | n.s. | n.s. | n.s. | n.s. | n.s. |
| F - T | n.s. | n.s. | n.s. | n.s. | n.s. | n.s. | -.645 | n.s. | n.s. |
| C - P | n.s. | n.s. | n.s. | n.s. | n.s. | n.s. | n.s. | n.s. | n.s. |
| F - P | n.s. | n.s. | n.s. | n.s. | n.s. | n.s. | -.625 | n.s. | n.s. |
| C - F | n.s. | n.s. | n.s. | n.s. | n.s. | n.s. | n.s. | n.s. | n.s. |

F=frontal; C=central; P=parietal; O=occipital; T=temporal; n.s.=not significant. The standardised regression coefficient β values are presented for P<0.05.

**P*<0.00056. Please note that significance at an alpha level of 0.05 involves the risk of a Type I error.

Table S15. Correlations between laterality indices (LIs) of coherence and reading ability calculated for children with ASD (*n*=26).The standardised regression coefficient β values for the multiple regression model are shown with the LIs calculated for each intrahemispheric coherence. The K-ABC subtest “Reading/Decoding” and age were applied as independent variables.

| Reading/  Decoding | Delta | Theta-1 | Theta-2 | Alpha-1 | Alpha-2 | Beta-1 | Beta-2 | Gamma-1 | Gamma-2 |
| --- | --- | --- | --- | --- | --- | --- | --- | --- | --- |
| T - O | n.s. | n.s. | n.s. | n.s. | .560 | n.s. | n.s. | n.s. | n.s. |
| P - O | n.s. | n.s. | n.s. | n.s. | n.s. | n.s. | n.s. | n.s. | n.s. |
| C - O | n.s. | n.s. | n.s. | n.s. | n.s. | n.s. | n.s. | n.s. | n.s. |
| F - O | n.s. | n.s. | .492 | n.s. | n.s. | n.s. | n.s. | n.s. | n.s. |
| P - T | n.s. | n.s. | n.s. | n.s. | n.s. | n.s. | -.537 | n.s. | -.820* |
| C - T | n.s. | n.s. | n.s. | n.s. | n.s. | n.s. | n.s. | n.s. | n.s. |
| F - T | n.s. | n.s. | n.s. | n.s. | .547 | n.s. | n.s. | -.643 | n.s. |
| C - P | n.s. | n.s. | n.s. | n.s. | n.s. | -.763 | n.s. | n.s. | n.s. |
| F - P | n.s. | n.s. | n.s. | n.s. | n.s. | n.s. | n.s. | n.s. | n.s. |
| C - F | n.s. | n.s. | n.s. | n.s. | n.s. | n.s. | n.s. | n.s. | n.s. |
| Age |  |  |  |  |  |  |  |  |  |
| T - O | n.s. | n.s. | n.s. | n.s. | -.579 | n.s. | n.s. | n.s. | n.s. |
| P - O | n.s. | n.s. | n.s. | n.s. | n.s. | n.s. | n.s. | n.s. | n.s. |
| C - O | n.s. | n.s. | n.s. | n.s. | n.s. | n.s. | n.s. | n.s. | n.s. |
| F - O | n.s. | n.s. | n.s. | n.s. | n.s. | n.s. | n.s. | n.s. | n.s. |
| P - T | n.s. | n.s. | n.s. | n.s. | n.s. | n.s. | n.s. | n.s. | .980* |
| C - T | n.s. | n.s. | n.s. | n.s. | n.s. | n.s. | n.s. | n.s. | .739 |
| F - T | n.s. | n.s. | n.s. | n.s. | n.s. | n.s. | n.s. | .526 | n.s. |
| C - P | n.s. | n.s. | n.s. | n.s. | n.s. | .640 | n.s. | n.s. | n.s. |
| F - P | n.s. | n.s. | n.s. | -.692 | n.s. | n.s. | n.s. | n.s. | n.s. |
| C - F | n.s. | n.s. | n.s. | n.s. | n.s. | n.s. | n.s. | n.s. | n.s. |

F=frontal; C=central; P=parietal; O=occipital; T=temporal; n.s.=not significant. The standardised regression coefficient β values are presented for P<0.05.

**P*<0.00056. Please note that significance at an alpha level of 0.05 involves the risk of a Type I error.

Table S16. Correlations between coherences in left hemisphere and reading ability calculated for TD children (*n*=26). The standardised regression coefficient β values for the multiple regression model are shown with intrahemispheric coherences in left hemisphere. The K-ABC subtest “Reading/Decoding” and age were applied as independent variables.

| Reading/  Decoding | Delta | Theta-1 | Theta-2 | Alpha-1 | Alpha-2 | Beta-1 | Beta-2 | Gamma-1 | Gamma-2 |
| --- | --- | --- | --- | --- | --- | --- | --- | --- | --- |
| T - O | n.s. | n.s. | n.s. | n.s. | n.s. | n.s. | n.s. | n.s. | n.s. |
| P - O | n.s. | n.s. | n.s. | n.s. | n.s. | n.s. | n.s. | n.s. | n.s. |
| C - O | n.s. | n.s. | n.s. | n.s. | n.s. | n.s. | n.s. | n.s. | n.s. |
| F - O | n.s. | n.s. | -.491 | -.621 | n.s. | n.s. | n.s. | n.s. | n.s. |
| P - T | n.s. | n.s. | n.s. | n.s. | n.s. | n.s. | n.s. | n.s. | n.s. |
| C - T | n.s. | n.s. | n.s. | n.s. | n.s. | n.s. | n.s. | n.s. | n.s. |
| F - T | -.562 | n.s. | n.s. | n.s. | n.s. | n.s. | n.s. | n.s. | n.s. |
| C - P | n.s. | n.s. | n.s. | n.s. | n.s. | n.s. | n.s. | n.s. | n.s. |
| F - P | n.s. | n.s. | n.s. | n.s. | n.s. | n.s. | n.s. | n.s. | n.s. |
| C - F | n.s. | n.s. | n.s. | n.s. | n.s. | n.s. | n.s. | n.s. | n.s. |
| Age |  |  |  |  |  |  |  |  |  |
| T - O | n.s. | .421 | n.s. | n.s. | n.s. | n.s. | n.s. | n.s. | n.s. |
| P - O | n.s. | n.s. | n.s. | n.s. | n.s. | n.s. | n.s. | n.s. | n.s. |
| C - O | n.s. | n.s. | n.s. | n.s. | n.s. | n.s. | .500 | n.s. | n.s. |
| F - O | n.s. | n.s. | n.s. | n.s. | n.s. | n.s. | n.s. | n.s. | n.s. |
| P - T | n.s. | n.s. | n.s. | n.s. | n.s. | n.s. | n.s. | n.s. | n.s. |
| C - T | n.s. | n.s. | n.s. | n.s. | n.s. | n.s. | .437 | n.s. | n.s. |
| F - T | n.s. | n.s. | n.s. | n.s. | n.s. | n.s. | n.s. | n.s. | n.s. |
| C - P | n.s. | n.s. | n.s. | n.s. | n.s. | n.s. | n.s. | n.s. | n.s. |
| F - P | n.s. | n.s. | n.s. | n.s. | n.s. | n.s. | n.s. | n.s. | n.s. |
| C - F | n.s. | n.s. | n.s. | n.s. | n.s. | n.s. | n.s. | n.s. | n.s. |

F=frontal; C=central; P=parietal; O=occipital; T=temporal; n.s.=not significant. The standardised regression coefficient β values are presented for P<0.05.

There was no significance for any interhemispheric coherence at an alpha level of 0.00056. Please note that significance at an alpha level of 0.05 involves the risk of a Type I error.

Table S17. Correlations between coherences in right hemisphere and reading ability calculated for TD children (*n*=26). The standardised regression coefficient β values for the multiple regression model are shown with intrahemispheric coherences in right hemisphere. The K-ABC subtest “Reading/Decoding” and age were applied as independent variables.

| Reading/  Decoding | Delta | Theta-1 | Theta-2 | Alpha-1 | Alpha-2 | Beta-1 | Beta-2 | Gamma-1 | Gamma-2 |
| --- | --- | --- | --- | --- | --- | --- | --- | --- | --- |
| T - O | n.s. | n.s. | n.s. | n.s. | n.s. | n.s. | .546 | n.s. | n.s. |
| P - O | n.s. | .536 | n.s. | n.s. | n.s. | n.s. | n.s. | n.s. | n.s. |
| C - O | n.s. | n.s. | n.s. | n.s. | n.s. | n.s. | n.s. | n.s. | n.s. |
| F - O | n.s. | n.s. | n.s. | n.s. | n.s. | n.s. | n.s. | n.s. | n.s. |
| P - T | n.s. | n.s. | n.s. | n.s. | n.s. | n.s. | n.s. | n.s. | n.s. |
| C - T | n.s. | n.s. | n.s. | n.s. | n.s. | n.s. | n.s. | n.s. | n.s. |
| F - T | n.s. | n.s. | n.s. | n.s. | n.s. | n.s. | n.s. | n.s. | n.s. |
| C - P | n.s. | n.s. | n.s. | n.s. | n.s. | n.s. | n.s. | n.s. | n.s. |
| F - P | n.s. | n.s. | n.s. | n.s. | n.s. | n.s. | n.s. | n.s. | n.s. |
| C - F | n.s. | .432 | n.s. | n.s. | n.s. | n.s. | n.s. | n.s. | n.s. |
| Age |  |  |  |  |  |  |  |  |  |
| T - O | n.s. | n.s. | n.s. | n.s. | n.s. | n.s. | n.s. | n.s. | n.s. |
| P - O | n.s. | n.s. | n.s. | n.s. | n.s. | n.s. | n.s. | n.s. | n.s. |
| C - O | n.s. | n.s. | n.s. | n.s. | n.s. | n.s. | n.s. | n.s. | n.s. |
| F - O | n.s. | n.s. | n.s. | n.s. | n.s. | n.s. | n.s. | n.s. | n.s. |
| P - T | n.s. | n.s. | n.s. | n.s. | n.s. | n.s. | n.s. | n.s. | n.s. |
| C - T | n.s. | n.s. | n.s. | n.s. | n.s. | n.s. | n.s. | n.s. | n.s. |
| F - T | n.s. | n.s. | n.s. | n.s. | n.s. | .506 | .434 | n.s. | n.s. |
| C - P | n.s. | n.s. | n.s. | n.s. | n.s. | n.s. | n.s. | n.s. | n.s. |
| F - P | n.s. | n.s. | n.s. | n.s. | n.s. | n.s. | n.s. | n.s. | n.s. |
| C - F | n.s. | n.s. | n.s. | n.s. | n.s. | n.s. | n.s. | n.s. | n.s. |

F=frontal; C=central; P=parietal; O=occipital; T=temporal; n.s.=not significant. The standardised regression coefficient β values are presented for P<0.05.

There was no significance for any interhemispheric coherence at an alpha level of 0.00056. Please note that significance at an alpha level of 0.05 involves the risk of a Type I error.

Table S18. Correlations between laterality indices (LIs) of coherences and reading ability calculated for TD children (*n*=26).The standardised regression coefficient β values for the multiple regression model are shown with the LIs calculated for each intrahemispheric coherence. The K-ABC subtest “Reading/Decoding” and age were applied as independent variables.

| Reading/  Decoding | Delta | Theta-1 | Theta-2 | Alpha-1 | Alpha-2 | Beta-1 | Beta-2 | Gamma-1 | Gamma-2 |
| --- | --- | --- | --- | --- | --- | --- | --- | --- | --- |
| T - O | n.s. | n.s. | n.s. | n.s. | n.s. | n.s. | n.s. | n.s. | n.s. |
| P - O | n.s. | n.s. | n.s. | n.s. | n.s. | n.s. | n.s. | n.s. | n.s. |
| C - O | n.s. | n.s. | n.s. | n.s. | n.s. | n.s. | n.s. | n.s. | n.s. |
| F - O | n.s. | n.s. | -.685* | -.468 | n.s. | n.s. | n.s. | n.s. | n.s. |
| P - T | n.s. | n.s. | n.s. | n.s. | n.s. | n.s. | n.s. | n.s. | n.s. |
| C - T | n.s. | n.s. | n.s. | n.s. | n.s. | n.s. | n.s. | n.s. | n.s. |
| F - T | -.515 | n.s. | n.s. | n.s. | n.s. | n.s. | n.s. | n.s. | n.s. |
| C - P | n.s. | n.s. | n.s. | n.s. | n.s. | n.s. | n.s. | n.s. | n.s. |
| F - P | n.s. | n.s. | n.s. | n.s. | n.s. | n.s. | n.s. | n.s. | n.s. |
| C - F | n.s. | n.s. | n.s. | n.s. | n.s. | n.s. | n.s. | n.s. | .437 |
| Age |  |  |  |  |  |  |  |  |  |
| T - O | n.s. | n.s. | n.s. | n.s. | n.s. | n.s. | n.s. | n.s. | n.s. |
| P - O | n.s. | n.s. | n.s. | n.s. | n.s. | n.s. | n.s. | n.s. | n.s. |
| C - O | n.s. | n.s. | n.s. | n.s. | n.s. | n.s. | n.s. | n.s. | n.s. |
| F - O | n.s. | n.s. | n.s. | n.s. | n.s. | n.s. | n.s. | n.s. | n.s. |
| P - T | n.s. | n.s. | n.s. | n.s. | n.s. | n.s. | n.s. | n.s. | n.s. |
| C - T | n.s. | n.s. | n.s. | n.s. | n.s. | n.s. | .463 | n.s. | n.s. |
| F - T | n.s. | n.s. | n.s. | n.s. | n.s. | -.432 | -.469 | n.s. | n.s. |
| C - P | n.s. | n.s. | n.s. | -.427 | n.s. | n.s. | n.s. | n.s. | n.s. |
| F - P | n.s. | n.s. | n.s. | n.s. | n.s. | n.s. | n.s. | n.s. | n.s. |
| C - F | n.s. | n.s. | n.s. | n.s. | n.s. | n.s. | n.s. | .491 | n.s. |

F=frontal; C=central; P=parietal; O=occipital; T=temporal; n.s.=not significant. The standardised regression coefficient β values are presented for P<0.05.

**P*<0.00056. Please note that significance at an alpha level of 0.05 involves the risk of a Type I error.

**Table S19. The *t*-values of intrahemispheric coherence for each hemisphere between ASD (*n* = 26) and TD (*n* = 26) children**

| Left | Delta | Theta-1 | Theta-2 | Alpha-1 | Alpha-2 | Beta-1 | Beta-2 | Gamma-1 | Gamma-2 |
| --- | --- | --- | --- | --- | --- | --- | --- | --- | --- |
| T - O | n.s. | n.s. | n.s. | n.s. | n.s. | 2.36 | n.s. | 2.20 | 2.27 |
| P - O | n.s. | n.s. | n.s. | n.s. | n.s. | n.s. | n.s. | n.s. | n.s. |
| C - O | n.s. | n.s. | n.s. | n.s. | n.s. | n.s. | n.s. | 2.12 | 2.02 |
| F - O | n.s. | n.s. | n.s. | n.s. | n.s. | n.s. | n.s. | n.s. | n.s. |
| P - T | n.s. | n.s. | n.s. | n.s. | n.s. | n.s. | n.s. | n.s. | 2.10 |
| C - T | n.s. | n.s. | n.s. | n.s. | n.s. | n.s. | n.s. | n.s. | n.s. |
| F - T | n.s. | n.s. | n.s. | n.s. | n.s. | n.s. | n.s. | n.s. | n.s. |
| C - P | n.s. | n.s. | n.s. | n.s. | n.s. | n.s. | n.s. | n.s. | n.s. |
| F - P | n.s. | n.s. | n.s. | n.s. | n.s. | n.s. | n.s. | n.s. | n.s. |
| C - F | n.s. | n.s. | n.s. | n.s. | n.s. | n.s. | n.s. | n.s. | n.s. |
| Right | Delta | Theta-1 | Theta-2 | Alpha-1 | Alpha-2 | Beta-1 | Beta-2 | Gamma-1 | Gamma-2 |
| T - O | n.s. | n.s. | n.s. | n.s. | n.s. | 2.16 | n.s. | 3.88* | 3.03 |
| P - O | n.s. | n.s. | n.s. | n.s. | n.s. | n.s. | n.s. | n.s. | 2.84 |
| C - O | n.s. | n.s. | n.s. | n.s. | n.s. | n.s. | n.s. | n.s. | n.s. |
| F - O | n.s. | n.s. | n.s. | n.s. | 2.23 | 2.06 | n.s. | n.s. | n.s. |
| P - T | n.s. | n.s. | n.s. | n.s. | n.s. | n.s. | n.s. | 2.60 | n.s. |
| C - T | n.s. | n.s. | n.s. | n.s. | n.s. | n.s. | 2.27 | n.s. | n.s. |
| F - T | n.s. | n.s. | n.s. | n.s. | n.s. | n.s. | n.s. | n.s. | n.s. |
| C - P | n.s. | n.s. | n.s. | n.s. | n.s. | n.s. | n.s. | n.s. | n.s. |
| F - P | 2.29 | n.s. | n.s. | n.s. | n.s. | n.s. | n.s. | n.s. | n.s. |
| C - F | n.s. | n.s. | -2.22 | n.s. | n.s. | n.s. | n.s. | n.s. | n.s. |

Positive values represent greater coherence values in the ASD than in the TD group. The *t*-values are presented for P<0.05. ASD, autism spectrum disorder; TD, typically developing; F, frontal; C, central; P, parietal; O, occipital; T, temporal. *, *P* < 0.00056. Please note that significance at an alpha level of 0.05 involves the risk of a Type I error.
